# Supplementary material for: Telemonitoring starting in the emergency department as an alternative to acute hospital admission: A prospective pilot study focusing on patient preferences and first experience
Source: PLOS Digit Health. 2025 Jul 31;4(7):e0000962. doi: 10.1371/journal.pdig.0000962 (PMC12312925; doi:10.1371/journal.pdig.0000962)
Supplement: S1 Text — (DOCX) [file pdig.0000962.s001.docx]

**Supplemental File 1: Preference questionnaire (ED cohort)**

1. What is your current living situation?

- Community dwelling
- Care home
- Nursing home
- Other

1. Do you have a roommate? (yes, no)
2. Is there someone who can help you (partner/informal caretaker)? (yes, no)
3. Which communication devices do you own? (yes/no)

- Landline
- Mobile phone

With apps

With video calling

With e-mail

- Computer/tablet/laptop
- Internet connection

1. Can you operate these communication devices independently or do you need help?

(yes, with help, no)

- Landline
- Mobile phone

With apps

With video calling

With e-mail

- Computer/tablet/laptop
- Internet connection

1. Do you own medical instruments? (yes/no)

- Blood pressure monitor
- Oxygen saturation meter
- Thermometer
- Smartwatch

1. Can you operate these medical instruments independently or do you need help?

(yes, with help, no)

- Blood pressure monitor
- Oxygen saturation meter
- Thermometer
- Smartwatch

1. Do you search the internet to retrieve information on diseases or health? (yes, with help, no)
2. Do you use e-mail and if so, can you open a link? (yes, with help, no)
3. Do you use app(lications)? (yes, with help, no)
4. Can you download an app(lication)? (yes, with help, no)

Do you use DigiD to watch your health data in the hospital chart? (yes, with help, no)

1. Have you ever heard of home monitoring or do you have experience with home monitoring?

- No, never heard of
- Yes, heard of, but no experience
- Yes, I have experience (if yes: what kind of experience do you have?)

1. Do you think that it is wise to return home, considering your current condition (yes, no)
2. If you think that you can return home now, do you need home monitoring? (yes, maybe, no)
3. If you think it is not wise/possible to return home now, please indicate why:

|  | Yes | Maybe | No |
| --- | --- | --- | --- |
| I am too severely ill |  |  |  |
| I do not have enough options to contact my treatment team |  |  |  |
| I am not capable of informing my doctors on my condition |  |  |  |
| My partner/housemate cannot provide enough help |  |  |  |
| I would feel unsafe at home |  |  |  |
| The treatment that I need cannot be provided at home |  |  |  |
| Other |  |  |  |

1. Hypothetically, if you would return home with home monitoring (independent of your current condition), what is your preferred way of contacting the treatment team?

- By phone
- By e-mail
- By video calling
- Using an app(lication)

1. What are conditions to be met for home monitoring (irrespective of your current condition)

- Someone to watch over me
- Someone to help me do the vital sign measurements
- Someone to help me establishing contact
- Daily contact with my treatment team
- Guaranteed contact with my treatment team day and night if problems arise
- Guaranteed admission if problems arise
- Other:..

1. Would you like to try a home monitor today? (yes, maybe, no)

*This questionnaire is based on: Weijers J, Prins MLM, van Dam D, AcuteCare@Home Study G, van Nieuwkoop C, Alsma J, et al. Patients' Perspectives and Feasibility of Home Monitoring in Acute Care: The AcuteCare@Home Flash Mob Study. Telemed J E Health. 2024, and Pharos Quickscan digital skills. [Available from: https://www.pharos.nl/kennisbank/quickscan-digitale-vaardigheden/]*
