# Supplementary material for: Telemonitoring starting in the emergency department as an alternative to acute hospital admission: A prospective pilot study focusing on patient preferences and first experience
Source: PLOS Digit Health. 2025 Jul 31;4(7):e0000962. doi: 10.1371/journal.pdig.0000962 (PMC12312925; doi:10.1371/journal.pdig.0000962)
Supplement: S2 Text — (DOCX) [file pdig.0000962.s002.docx]

**Supplemental File 2: Assessment questionnaire for nurses and physicians**

1. Is it possible to avoid admission by using telemonitoring in this patient? (yes/no/maybe)
2. Do you think this patient is physically capable of returning home with telemonitoring (yes/no/maybe)
3. Do you think this patient is cognitively capable of returning home with telemonitoring (yes/no/maybe)
4. Do you think this patient’s home support is sufficient to return safely home with telemonitoring (yes/no/maybe)
