# Supplementary material for: Telemonitoring starting in the emergency department as an alternative to acute hospital admission: A prospective pilot study focusing on patient preferences and first experience
Source: PLOS Digit Health. 2025 Jul 31;4(7):e0000962. doi: 10.1371/journal.pdig.0000962 (PMC12312925; doi:10.1371/journal.pdig.0000962)
Supplement: S3 Text — (DOCX) [file pdig.0000962.s003.docx]

**Supplemental File 3: Experience questionnaire patients (telemonitor cohort)**

1. The explanations/instructions provided in the ED were clear and helped me using the telemonitor system (1-5; completely disagree – completely agree)
2. I have insight into the telemonitor system (1-5)
3. The plaster/patch was comfortable to wear (1-5)
4. The placement of the router was easy (1-5)
5. The router was well connected (1-5)
6. I found the system unnecessarily complex (1-5)*
7. I am satisfied with the plaster
8. I am satisfied with the router
9. I am satisfied with this telemonitor system (1-5)
10. I think this system was easy-to-use (1-5)*
11. I felt very confident using this system (1-5)*
12. I needed to learn a lot of things before I could get going with this system (1-5)*
13. I think I want to use this system more frequently (1-5)*
14. I experienced no problems with my privacy (1-5)
15. This telemonitor system made me feel safe (1-5)
16. This system offers a good alternative to admission (1-5)
17. I would prefer to measure vitals myself (1-5)
18. I would recommend this telemonitoring system to family and friends (1-5)

Based, in part, on the SUS questionnaire: *Ensink CJ, Keijsers NLW, Groen BE. Translation and validation of the System Usability Scale to a Dutch version: D-SUS. Disabil Rehabil. 2024;46(2):395-400.*
