# Supplementary material for: Telemonitoring starting in the emergency department as an alternative to acute hospital admission: A prospective pilot study focusing on patient preferences and first experience
Source: PLOS Digit Health. 2025 Jul 31;4(7):e0000962. doi: 10.1371/journal.pdig.0000962 (PMC12312925; doi:10.1371/journal.pdig.0000962)
Supplement: S4 Text — (DOCX) [file pdig.0000962.s004.docx]

**Supplemental File 4: Experience questionnaire investigators**

1. The telemonitor system was easy to install

(1-5; completely disagree – completely agree)

2. The information yielded by tele monitoring system was easy to follow (1-5)

3. The telemonitor system was easy to handle by the patients (1-5)

4. Vital signs alarms come through (1-5)

5. Vital sign alarms are justified (1-5)

6. Technical alarms come through (1-5)

7. Technical issues can be solved (1-5)

8. This telemonitor system is fit for home monitoring (1-5)

9. Real time continuous monitoring is needed for a virtual ward (1-5)
