# Supplementary material for: Telemonitoring starting in the emergency department as an alternative to acute hospital admission: A prospective pilot study focusing on patient preferences and first experience
Source: PLOS Digit Health. 2025 Jul 31;4(7):e0000962. doi: 10.1371/journal.pdig.0000962 (PMC12312925; doi:10.1371/journal.pdig.0000962)
Supplement: S1 Table — (DOCX) [file pdig.0000962.s005.docx]

**Supplemental Table 1: ED main diagnoses telemonitorcohort**

| **ED diagnosis** | **Number of patients** | **Number of hospital admissions** |
| --- | --- | --- |
| Influenza A | 3 | 2 |
| Urosepsis | 1 | 1 |
| Pneumonia | 1 | 1 |
| Skin infection | 1 | 1 |
| Gastroenteritis | 1 | 0 |
| Hypertension | 3 | 0 |
| Syncope | 2 | 0 |
| Heartfailure | 1 | 1 |
| Thromboflebitis leg | 1 | 0 |
| Anemia | 2 | 2 |
| Acute renal failure | 1 | 1 |
| Metabolic acidosis | 1 | 0 |
| Other |  |  |
| Suspected VTE* | 1 | 0 |
| Suspected electrolyt disturbance* | 1 | 0 |
| Suspected systemic vasculitis* | 1 | 0 |

*suspected diagnosis was not confirmed
