# Supplementary material for: Telemonitoring starting in the emergency department as an alternative to acute hospital admission: A prospective pilot study focusing on patient preferences and first experience
Source: PLOS Digit Health. 2025 Jul 31;4(7):e0000962. doi: 10.1371/journal.pdig.0000962 (PMC12312925; doi:10.1371/journal.pdig.0000962)
Supplement: S2 Table — (DOCX) [file pdig.0000962.s006.docx]

**Supplemental Table 2: Availability of and proficiency with devices and instruments, and digital skills**

|  | **ED cohort**  **N=98** | | **Telemonitor cohort**  **N=21** | |
| --- | --- | --- | --- | --- |
|  | **Availability** | **Independent use** | **Availability** | **Independent use** |
| **Communication devices** |  |  |  |  |
| Phone; landline | 47 (48.0) | 96 (98.0) | 8 (38.1) | 20 (95.2) |
| Phone; mobile | 93 (94.9) | 88 (89.8) | 21 (100.0) | 19 (90.5) |
| Video calling | 69 (70.4) | 65 (66.3) | 14 (66.7) | 13 (61.9) |
| E-mail | 75 (76.5) | 68 (69.4) | 17 (81.0) | 14 (66.7) |
| Computer/laptop/tablet | 80 (81.6) | 70 (71.4) | 20 (95.2) | 14 (66.7) |
| Internet | 86 (87.8) | 71 (72.4) | 20 (95.2) | 16 (76.2) |
| **Medical instruments** |  |  |  |  |
| Blood pressure monitor | 55 (56.1) | 55 (56.1) | 15 (71.4) | 16 (76.2) |
| Oxygen saturation meter | 19 (19.4) | 46 (46.9) | 3 (14.3) | 11 (52.4) |
| Thermometer | 88 (89.8) | 89 (90.8) | 18 (85.7) | 19 (90.5) |
| Smartwatch | 18 (18.4) | 18 (18.4) | 4 (19.0) | 5 (23.8) |
| **Digital skills** |  |  |  |  |
| Search about health on the internet |  | 59 (60.2) |  | 12 (57.1) |
| Open link in e-mail |  | 62 (63.3) |  | 14 (66.7) |
| Use apps |  | 69 (70.4) |  | 16 (76.2) |
| Download apps |  | 58 (59.2) |  | 14 (66.7) |
| Use of DigiD |  | 69 (70.4) |  | 15 (71.4) |
| **Digital skills** |  |  |  |  |
| Not skilled-beginner |  | 28 (28.6) |  | 6 (28.6) |
| Moderately skilled |  | 7 (7.1) |  | 2 (9.5) |
| Advanced |  | 63 (64.3) |  | 13 (61.9) |
| **Experience with telemonitoring** |  |  |  |  |
| No | 62 (63.3) |  |  | 9 (42.9) |
| Heard of | 29 (29.6) |  |  | 7 (33.3) |
| Yes | 7 (7.1) |  |  | 5 (23.8) |
