# Supplementary material for: Telemonitoring starting in the emergency department as an alternative to acute hospital admission: A prospective pilot study focusing on patient preferences and first experience
Source: PLOS Digit Health. 2025 Jul 31;4(7):e0000962. doi: 10.1371/journal.pdig.0000962 (PMC12312925; doi:10.1371/journal.pdig.0000962)
Supplement: S3 Table — (DOCX) [file pdig.0000962.s007.docx]

**Supplemental Table 3: Inclusion bias analysis**

|  | **Included patients**  **N=98** | **Non-included patients**  **N=21** | **P value** |
| --- | --- | --- | --- |
| **Age** | 66.3 (16.6) | 64.8 (16.7) | 0.69 |
| **Male** | 49 (50.0) | 10 (47.6) | 1.0 |
| **Triage code (MTS)** |  |  | 0.48 |
| Blue | 2 (2.0) | 0 |  |
| Green | 36 (36.7) | 9 (42.9) |  |
| Yellow | 44 (44.9) | 10 (47.6) |  |
| Orange | 15 (15.3) | 1 (4.8) |  |
| Red | 1 (1.0) | 1 (4.8) |  |
| **Discharge destination** |  |  | 0.07 |
| Regular ward | 51 (52.0) | 14 (66.7) |  |
| Medium care unit | 3 (3.1) | 0 |  |
| Intensive care unit | 0 | 1 (4.8) |  |
| Home | 44 (44.9) | 6 (28.6) |  |
