# Supplementary material for: Telemonitoring starting in the emergency department as an alternative to acute hospital admission: A prospective pilot study focusing on patient preferences and first experience
Source: PLOS Digit Health. 2025 Jul 31;4(7):e0000962. doi: 10.1371/journal.pdig.0000962 (PMC12312925; doi:10.1371/journal.pdig.0000962)
Supplement: S4 Table — (DOCX) [file pdig.0000962.s008.docx]

**Supplemental Table 4: Agreement between patient and nurse on assessment of patient capability to use telemonitoring**

|  | **Nurse:**  **Patient fully**  **capable** | **Nurse:**  **Patient not fully capable** |  |
| --- | --- | --- | --- |
| **Patient fully capable** | 38 | 8 | 46 |
| **Patient not fully capable** | 18 | 32 | 50 |
|  | 54 | 42 | 96 |

Kappa 0.161; 2 assessments missing
