# Supplementary material for: Telemonitoring starting in the emergency department as an alternative to acute hospital admission: A prospective pilot study focusing on patient preferences and first experience
Source: PLOS Digit Health. 2025 Jul 31;4(7):e0000962. doi: 10.1371/journal.pdig.0000962 (PMC12312925; doi:10.1371/journal.pdig.0000962)
Supplement: S5 Table — (DOCX) [file pdig.0000962.s009.docx]

**Supplemental Table 5: Alarms in telemonitorcohort**

|  | **Number of alarms** | **Number of patients** |
| --- | --- | --- |
| **Total** | 90 | 17 |
| **Low temperature** | 45 | 12 |
| Misplacement temperature lead | 44 |  |
| **High temperature** | 11 | 6 |
| **Respiratory rate** | 29 | 7 |
| **Heart rate** | 5 | 3 |
