# Supplementary material for: Telemonitoring starting in the emergency department as an alternative to acute hospital admission: A prospective pilot study focusing on patient preferences and first experience
Source: PLOS Digit Health. 2025 Jul 31;4(7):e0000962. doi: 10.1371/journal.pdig.0000962 (PMC12312925; doi:10.1371/journal.pdig.0000962)
Supplement: Attachment — Submitted filename: PDIG-D-25-00071_reviewer.pdf [file pdig.0000962.s011.pdf]

// PLOS Digital Health

Telemonitoring at home as alternative for admission in acute care. A prospective pilot study focusing on patient perspectives and first experiences

--Manuscript Draft--

**Manuscript Number:**	PDIG-D-25-00071
**Article Type:**	Research Article
**Full Title:**	Telemonitoring at home as alternative for admission in acute care. A prospective pilot study focusing on patient perspectives and first experiences
**Short Title:**	Telemonitoring in acute care
**Corresponding Author:**	Patricia M Stassen<br>Maastricht University Hospital: Maastricht Universitair Medisch Centrum+<br>Maastricht, Limburg NETHERLANDS, KINGDOM OF THE
**Order of Authors:**	Noortje Zelis, PhD
	Dewa Westerman
	Anouk Schevers
	Nicole v Eldik, PhD
	Patricia M Stassen, PhD
**Keywords:**	Telemonitoring; Admission avoidance; Admission alternative; Acute care; Emergency Department
**Abstract:**	Introduction<br><br>Telemonitoring at home may be used to reduce admissions via the emergency department (ED), but experience in this setting is scarce. We performed a pilot study to investigate the perspectives and experiences of patients and care professionals with telemonitoring.<br><br>Methods<br><br>In this prospective pilot study, we asked medical ED patients on their perspectives on home monitoring. In a subset, we started and evaluated telemonitoring.<br><br>Results<br><br>In total, 98 patients answered the questionnaire. The prerequisites for telemonitoring were guaranteed admission if necessary (96.9%), possibility to contact the treatment team 24/7 (90.8%) and presence of someone to watch over the patient (72.4%). Main barriers were: treatment could not be provided at home, feeling too ill, and judging it unsafe to return home.<br>In total, 11.2% of patients indicated that admission could be avoided using telemonitoring, while 6.1% thought this might be possible. For professionals, these proportions were lower, and agreement with patients was slight-fair.<br>All telemonitored patients were satisfied with the easiness of use and comfortability of the system, which gave most patients a reassuring feeling and was considered an alternative for admission..<br><br>Conclusion<br><br>Telemonitoring at home was seen as alternative for admission in a substantial proportion of medical ED patients. Prerequisites were guaranteed admission and 24/7 contact with the treatment team, while barriers were related to disease severity and lack of someone to watch over the patient. Telemonitoring can be used in acute care as alternative for admissions if prerequisites are met and barriers removed.
**Additional Information:**	

Powered by Editorial Manager® and ProduXion Manager® from Aries Systems Corporation

Question	Response
**Government Employee**<br><br>Are you or any of the contributing authors an employee of the United States government?<br><br>Manuscripts authored by one or more US Government employees are not copyrighted, but are licensed under a CC0 Public Domain Dedication, which allows unlimited distribution and reuse of the article for any lawful purpose. This is a legal requirement for US Government employees.<br><br>This will be typeset if the manuscript is accepted for publication.	No - No authors are employees of the U.S. government.
**Financial Disclosure**<br><br>Enter a financial disclosure statement that describes the sources of funding for the work included in this submission. Review the submission guidelines for detailed requirements. View published research articles from *PLOS Digital Health* for specific examples.<br><br>This statement is required for submission and **will appear in the published article** if the submission is accepted. Please make sure it is accurate.<br><br>**Funded studies**<br>Enter a statement with the following details:<br>• Initials of the authors who received each award<br>• Grant numbers awarded to each author<br>• The full name of each funder<br>• URL of each funder website<br>• Did the sponsors or funders play any role in the study design, data collection and analysis, decision to publish, or preparation of the manuscript?	The author(s) received no specific funding for this work.

Did you receive funding for this work?	
**Competing Interests**<br><br>On behalf of all authors, disclose any competing interests that could be perceived to bias this work.<br><br>This statement will be typeset if the manuscript is accepted for publication.<br><br>Review the instructions link below and PLOS Digital Health's competing interests policy to determine what information must be disclosed at submission.	None
**Data Availability**<br><br>Provide a **Data Availability Statement** in the box below. This statement should detail where the data used in this submission can be accessed. This statement will be typeset if the manuscript is accepted for publication.<br><br>Before publication, authors are required to make all data underlying their findings fully available, without restriction. Review our PLOS Data Policy page for detailed information on this policy. Instructions for writing your Data Availability statement can be accessed via the Instructions link below.	all data underlying their findings fully available, without restriction

Powered by Editorial Manager® and ProduXion Manager® from Aries Systems Corporation

Manuscript

Click here to access/download;Manuscript;telemonitoring acute care main manuscript.docx

Telemonitoring at home as alternative for admission in acute care. A prospective pilot study focusing on patient perspectives and first experiences

Noortje Zelis[1,2], Dewa Westerman[1], Anouk Schevers[3], Nicole v Eldik[3,4], Patricia M. Stassen[1,2]

[1] Department of Internal Medicine, division general medicine, section acute medicine, Maastricht University Medical Center

[2] Cardiovascular Research Institute Maastricht (CARIM), Maastricht University

[3] Healthcare Innovation Lab, Maastricht University Medical Center

[4] Department of Pediatrics, Maastricht University Medical Center

Abstract

Introduction

Telemonitoring at home may be used to reduce admissions via the emergency department (ED), but experience in this setting is scarce. We performed a pilot study to investigate the perspectives and experiences of patients and care professionals with telemonitoring.

Methods

In this prospective pilot study, we asked medical ED patients on their perspectives on home monitoring. In a subset, we started and evaluated telemonitoring.

Results

In total, 98 patients answered the questionnaire. The prerequisites for telemonitoring were guaranteed admission if necessary (96.9%), possibility to contact the treatment team 24/7 (90.8%) and presence of someone to watch over the patient (72.4%). Main barriers were: treatment could not be provided at home, feeling too ill, and judging it unsafe to return home.

In total, 11.2% of patients indicated that admission could be avoided using telemonitoring, while 6.1% thought this might be possible. For professionals, these proportions were lower, and agreement with patients was slight-fair.

All telemonitored patients were satisfied with the easiness of use and comfortability of the system, which gave most patients a reassuring feeling and was considered an alternative for admission..

Conclusion

Telemonitoring at home was seen as alternative for admission in a substantial proportion of medical ED patients. Prerequisites were guaranteed admission and 24/7 contact with the treatment team, while barriers were related to disease severity and lack of someone to watch over the patient.

Telemonitoring can be used in acute care as alternative for admissions if prerequisites are met and barriers removed.

Keywords:

Telemonitoring

Admission avoidance

Admission alternative

Acute care

Emergency department

Introduction

In order to relieve the increasing pressure on the healthcare system and to adjust to preferences of patients, alternative forms of care are being explored. One of these alternatives is telemonitoring at home. By monitoring specific aspects of health and disease, patients can manage their health or disease within their own environment. Continuous telemonitoring of vital signs has proven practical, and is well-received, safe and effective in decreasing care demands for a number of chronic conditions (1-4). Whether these findings in chronic conditions can be extrapolated to acute conditions is currently unclear. The acute care setting differs from the chronic setting, and patients who visit the emergency department (ED) are likely to respond differently than patients with chronic conditions. Adapting to new instruments and a new form of care is likely to be challenging.

To date, experience with telemonitoring in acute conditions in acute settings is scarce. Most experience stems from the COVID-19 pandemic (5-8). As alternative for admission, patients were sent home with oxygen, medication and instruments to measure vital signs. Telemonitoring at home turned out feasible and safe, and many patients could be treated outside the hospital. However, there is limited experience with other acute conditions (9-13) The results were promising, and the patients recovered faster and moved more at home than patients who were admitted (12).

This limited experience with home monitoring in acute conditions warrant more studies. Before implementation on a larger scale, however, patient experience with home monitoring devices and their preferences should be further explored, as are the ways to select patients who are suitable for and capable of using home monitoring (9, 14, 15). If more information on these aspects becomes available, the necessary steps can be made to implement a new form of acute care starting in the ED.

We therefore conducted a prospective pilot study in order to investigate the experiences of medical patients and acute care physicians with continuous telemonitoring started in the setting of an ED. We also investigated the preferences of ED patients regarding telemonitoring as continued care. In addition, we aimed to retrieve the proportion of patients who could be treated at home instead of in the hospital by using telemonitoring.

Methods

Design/setting

This prospective pilot study was performed in the ED of Maastricht University Medical Centre, which provides secondary and tertiary care. In the ED, about 7000 patients are assessed and treated by (acute) internists and their residents per year. On average, two thirds of these ED patients are admitted to the hospital.

In the study period, ED patients and their treating physicians and nurses were asked to complete questionnaires on home monitoring, and a subgroup of patients was then asked to try a telemonitoring system for at least 24 hours.

Patients

All adult (18+) ED patients who were assessed and treated by internists during 10 consecutive weekdays between 7:30 and 15:30h, were asked informed consent and, if provided, included in the study. Exclusion criteria were: an ED revisit (patients could be included only once during the study period), being too severely ill or confused to answer the questions in a reliable way without putting too much strain on patient or family, and a language barrier. All patients who provided consent were asked to complete a questionnaire (ED cohort).

Part of the ED patients formed a second cohort (telemonitor cohort). They were asked additional consent to wear a telemonitoring system for at least 24 hours. Patients were eligible for this cohort if they lived within a 15 km radius of the hospital. In addition, they were either about to be sent home (i.e. judged not in need for admission), or were admitted to the hospital without requiring continuous monitoring of vital signs (i.e. not in need of telemonitoring or admission to either a medium or intensive care unit (MCU/ICU, resp.). The telemonitor patients who returned home were asked to measure their vital signs (blood pressure, heartrate, oxygen saturation and temperature) three times a day.

Telemonitoring system

For this study, a telemonitoring device consisting of a plaster, applied to the chest, and a lead guided through the axilla of the non-dominant arm. The plaster and lead continuously

measure the respiratory rate, heartrate, axilla temperature, and generate an output every 2 minutes. The thresholds of the vital signs and of the period during which the thresholds before an alarm was raised were agreed in advance. The data were transmitted by a portable router, which in turn transmitted the measurements and alarms via a web application.

Neither patients nor hospital staff were aware of the results of the measurements. If the vital signs or alerts prompted further investigation or action, two of the investigators (experienced acute internists) contacted the treatment team (admitted patients) or patients (at home).

Data collection and procedures

For the ED cohort, a *preference questionnaire* was used (Supplemental file 1, in part based on (16, 17)), and filled in by all participants at the end of their ED visit. The questionnaire aimed to retrieve data on the availability of and skills regarding communication, medical and digital instruments (e.g. blood pressure monitor and digital skills {Anonymous, #4}). In addition, preferences were retrieved regarding the prerequisites to be fulfilled in order to return home. Furthermore, questions were asked on the judgement of their ability to return home in their current condition, and, when applicable, on the reasons why returning home was not possible.

For the ED cohort, physicians and nurses filled out a short *assessment questionnaire* on the potential of home monitoring as alternative for admission, and on the capabilities of patients to use home monitoring in three domains: physical, cognitive, home support (Supplemental file 2).

For the telemonitor cohort, an *experience questionnaire* regarding patient experience with telemonitoring was completed at the end of their monitoring period (Supplemental file 3), in part based on the SUS questionnaire (18). For this cohort, data generated by the telemonitor system were retrieved, and generated alarms were compared with either vital sign measurements during admission (measured as usual by nurses) or by self-measurements of the vital signs three times a day or vice versa (aberrant vital signs that were manually measured were compared with the monitor data).
The experience with the telemonitoring system of the two acute internists was retrieved at the end of the pilot using a short *experience questionnaire* (Supplemental file 4).

Electronic medical charts were used to collect data on age, sex, means of transportation to the ED, triage category (using MTS: low (blue, green) or highly urgent (yellow/orange/red) (19)), vital signs (necessary to calculate MEWS scores (20)), ED diagnosis (categorized according ICD-10 (21)), treatment started at the ED (oxygen yes/no, infusion yes/no, intravenous antibiotics yes/no), ED discharge destination (general ward, MCU/ICU, home), and length of hospital stay.

Outcome measures

Primary

The proportion of patients satisfied with telemonitoring (telemonitor cohort)

Secondary

Proportion of patients willing and able to return home from the ED with telemonitoring as alternative for admission

Agreement between by patient, physician and nurse assessments of being “fully capable” (physical, cognitive and home support) for telemonitoring and for avoiding admission.

The number and nature of generated alarms.

Analysis and statistics

The main part of the analyses regarding the experience with telemonitoring and patient preferences were merely descriptive; for patient characteristics, preferences, and experiences, means (with SD), medians (with IQR) or proportions were calculated. Comparisons were made using Student T tests, Chi2 tests or Mann Whitney U tests, where appropriate. In case of missing data, valid percentages were calculated.

Agreement on judgment of being capable for returning home with telemonitoring between patients, physicians and nurses were then analysed by calculating Cohen’s kappa, whereas a kappa of 0-0.20 indicates slight, 0.21-0.40 fair, 0.41-0.60 moderate, 0.61-0.80 substantial, and >0.81 almost perfect agreement (22).

The number and nature of generated alerts were described and compared with measurements (self or in-hospital) of vital signs, in a descriptive way. No formal statistical agreement could be determined because 1) the measurements generated by the telemonitoring system focus on trends, rather than on individual measurements of vital signs, 2) vital signs were only

measured 3 times a day or on indication (admitted patients), and therefore often not measured at the same time as the generated alerts, 3) patients did not measure their respiratory rate (non-admitted patients).

We used IBM SPSS Statistics for Windows, version 28.0 (IBM Corp., Armonk, N.Y., USA) for the statistical analysis. P values <0.05 were considered statistically significant.

Ethics

This study was approved by the Medical Ethics Committee of MUMC+ (METC approval: 2023-0248). Written informed consent was provided by all patients.

Results

Study population

In the study period, a total of 119 patients presented to the ED and were assessed and treated by internists (Figure 1). Of these 21 were excluded. In total, 98 patients answered the questionnaire and formed the ED cohort, while 21 of these patients agreed to try the telemonitor system and formed the Telemonitor cohort. Of these, 12 returned home and 9 were admitted to the hospital.

Figure 1 Flowchart

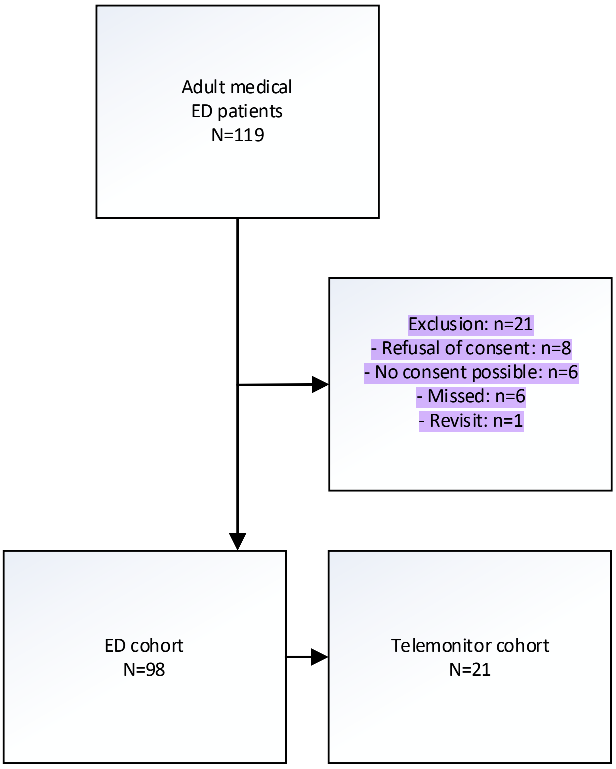

The median age of the 98 patients was 68.5 (IQR: 53-78.3; Table 1). All but one were community dwelling, and 65.3% were living with a roommate. The majority of patients were digitally skilled (advanced: 64.3%). In total, 54 (55.1%) patients were admitted to the hospital. The vast majority of these patients were treated with oxygen, and/or with intravenous fluid or medication.8

Table 1: Baseline characteristics

N (%) or median (IQR)	ED cohort N=98	Telemonitor cohort N=21
**Demographics**		
Age	68.5 (53-78.3)	70 (60.0-76.5)
≥75 years	35 (35.7)	8 (38.1)
Male	49 (50.0)	12 (57.1)
**Living situation**		
Community dwelling	97 (99.0)	20 (95.2)
Roommate	64 (65.3)	14 (66.7)
Informal caretaker	82 (83.7)	18 (85.7)
**Digital skills**		
Not skilled-beginner	28 (28.6)	6 (28.6)
Moderately skilled	7 (7.1)	2 (9.5)
Advanced	63 (64.3)	13 (61.9)
**Acute care data/disease severity**		
Transportation by ambulance	17 (17.3)	4 (19.0)
Triage code (MTS)		
Blue/green	38 (38.8)	11 (52.4)
Yellow/orange/red	60 (61.2)	10 (47.6)
MEWS	1 (0-9)	2 (0-5)
MEWS≥3	28 (28.6)	8 (38.1)
**Subspecialty**		
General internal medicine	52 (53.1)	17 (81.0)
Gastroenterology	19 (19.4)	0
Oncology	14 (14.3)	0
Haematology	4 (4.1)	1 (4.8)
Immunology	4 (4.1)	1 (4.8)
Nephrology	3 (3.1)	1 (4.8)
Other	2 (2.0)	1 (4.8)
**ED diagnosis/ICD-10**		
Infectious diseases	36 (36.7)	7 (33.3)
Digestive system	17 (17.3)	0
Cardiovascular system	14 (14.3)	7 (33.3)
Blood/blood forming organs	12 (12.2)	2 (9.5)
Genitourinary system	6 (6.1)	1 (4.8)
Neoplasm	4 (4.1)	0
Endocrine/metabolic	3 (3.1)	1 (4.8)
Other	6 (6.1)	3 (14.3)
**Continued care**		
Admission to hospital	54 (55.1)	9 (42.9)
Regular ward	51 (52.0)	9 (42.9)
Medium care unit	3 (3.1)	0

Length of hospital-stay (days)	4 (1-58)	5 (1-58)
**Initial treatment after admission (n=54; n=9)**		
Oxygen	14 (25.9)	3 (33.3)
Intravenous fluids	47 (87.0)	9 (100.0)
Intravenous antibiotics	23 (42.6)	5 (55.6)
Oxygen or intravenous fluids/medication	49 (90.7)	9 (100.0)

There were no significant differences in age, sex, triage code nor in discharge destination between included and non-included patients (Supplemental Table 2).

Almost all patients had a mobile phone and a slight majority had medical instruments and were able to use these independently (Supplemental Table 1). Only a few (n=7; 7.1%) patients had experience with telemonitoring.

Patient preferences

Most patients preferred the phone as a way of contacting the treatment team (Table 2). The three most frequently mentioned prerequisites for telemonitoring were guaranteed admission if necessary (96.9%), possibility to contact the treatment team 24/7 (90.8%) and the presence of someone to watch over/assist the patient (72.4%).

In total, 51 (52.0%) patients indicated that they were not able to go home considering their current condition (Table 2). The main reasons were the judgement that the necessary treatment could not be provided at home, feeling too severely ill, and judging it unsafe to go home.

Table 2: Patient preferences

	ED cohort (all patients) N=98	Telemonitor cohort N=21
**Preferred way of communication with professionals**		
Phone	74 (75.5)	17 (81.0)
E-mail	5 (5.1)	0
Video calling	15 (15.3)	3 (14.3)
App	4 (4.1)	1 (4.8)
**Prerequisites for telemonitoring**		
Guaranteed admission if necessary	95 (96.9)	21 (100.0)
Possibility to contact treatment team 24/7	89 (90.8)	18 (85.7)
Someone to watch over me	71 (72.4)	12 (57.1)
Help with the measurements	46 (46.9)	9 (42.9)
Daily contact with the treatment team	42 (42.9)	9 (42.9)
Help in establishing contact with treatment team	30 (30.6)	5 (23.8)
**Reasons for not being able to go home (n=51)**		
Treatment not possible at home	50 (98.0)	
Too severely ill	42 (82.3)	
Unsafe to go home	29 (56.9)	
Insufficient help at home	13 (25.5)	
Not able to inform about my condition	8 (15.7)	
Not able to establish contact	5 (9.8)	

Home monitoring as alternative for admission

Of 98 patients, 11 (11.2%) indicated that an admission could be avoided by using telemonitoring, while another 6 (6.1%) thought that this might be possible (Table 3). For professionals, these proportions were somewhat lower, and agreement between patients and either physicians or nurses regarding avoidance of admission was slight-fair with kappa values of 0.240 and 0.210, respectively. Agreement between physician and nurse was moderate (kappa 0.539) for all patients, and for those who were admitted (Supplemental Tables 3A and B).

Table 3: Avoidance of admission by telemonitoring: three perspectives**

N (%)	**Patient**	**Physician**	**Nurse**
Avoid admission: no	51 (52.0)	49 (50.5)	47 (49.0)
Avoid admission: yes	11 (11.2)	7 (7.2)	10 (10.4)
Avoid admission: maybe	6 (6.1)	6 (6.1)	4 (4.2)
Home without telemonitoring	30 (30.6)	35 (36.1)	35 (36.5)
*Missing***		1	2

* Valid percentages were calculated, ** Agreement after exclusion of category “home without telemonitoring”. Kappa values: patient-physician: 0.240; patient-nurse: 0.210; physician-nurse: 0.539

Assessment of capability to use telemonitoring

In total, physicians judged 47 of 97 patients (48.5%) to be capable to use telemonitoring with regard to the three domains (physical, cognitive and home support; Table 4). The agreement with the patients’ perspective was slight with a kappa of 0.153. Agreement between physicians and nurses was almost perfect (kappa 0.872, data not shown). Agreement between nurses and patients was slight with a kappa of 0.161 (data not shown).

Table 4: Agreement between patient and physician of ability to use telemonitoring

	**Physician: Patient fully capable**	**Physician: Patient not fully capable**	
**Patient: capable**	10	37	47
**Patient: not capable**	33	17	50
	47	54	97

kappa = 0.153; 1 missing

Experience with telemonitoring – patient perspective

In total, 21 patients used the telemonitor system. Their details are depicted in Table 1. Of these, 9 were admitted to the hospital. The other 12 patients were discharged home. The process of explaining and connecting the system to the patients took about fifteen to twenty minutes per patient. At the end of their monitoring episode, all patients reported to be satisfied or very satisfied with the instruction and explanation (insight), the easiness of use of the telemonitor system, the placement of the router, and all but one (95.2%) were (very) self-confident in using the system (Figure 2A and B). Most patients (90.5%) judged the plaster to be (very) comfortable.

Figure 2: Patient experience with telemonitoring

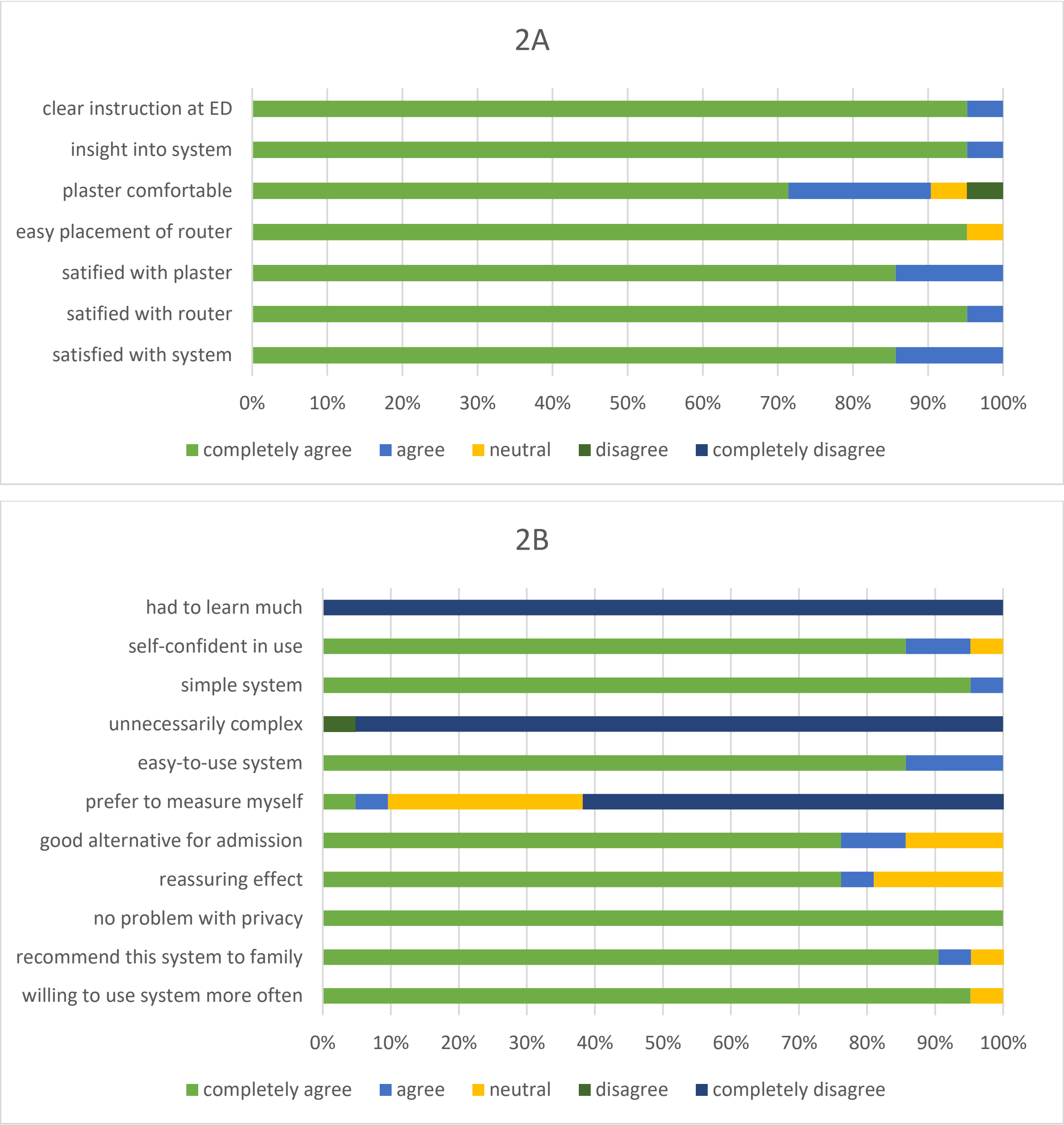

Figure 2A: Instruction and set-up
Figure 2B: Experience with telemonitoring

The concept of telemonitoring gave most patients (81.0%) a (very) reassuring feeling and none experienced problems with their privacy (Figure 3). All patients indicated to be willing to use the system again, and would recommend it to their family or friends. All but three (85.7%) considered telemonitoring a good alternative for admission. In contrast, most patients (61.9%) indicated that they would not prefer to do the measurement their selves.

Experience with telemonitoring - physician perspective

The telemonitoring system was easy to install at the ED, and patients could be quickly instructed (Figure 3). It was easy to follow the patients from a distance; both the vital signs and technical alarms. It was however difficult to assist a patient from a distance when technical problems arose (e.g. detachment of the plaster or displacement of the temperature lead).

In total, 90 alarms were raised in 17 patients (median: 4 per patient). Half (n=45,48.9%) of these were alerts of a low body temperature (in 12 patients). All but one of these alarms could be explained by misplacement of or a misplaced temperature lead. Other alerts were high temperature (n=11; confirmed in 10), high respiratory rate (n=29; confirmed in 19; no self-measurements in 10) and high heart rate (n=5).

Figure 3: Physician experience

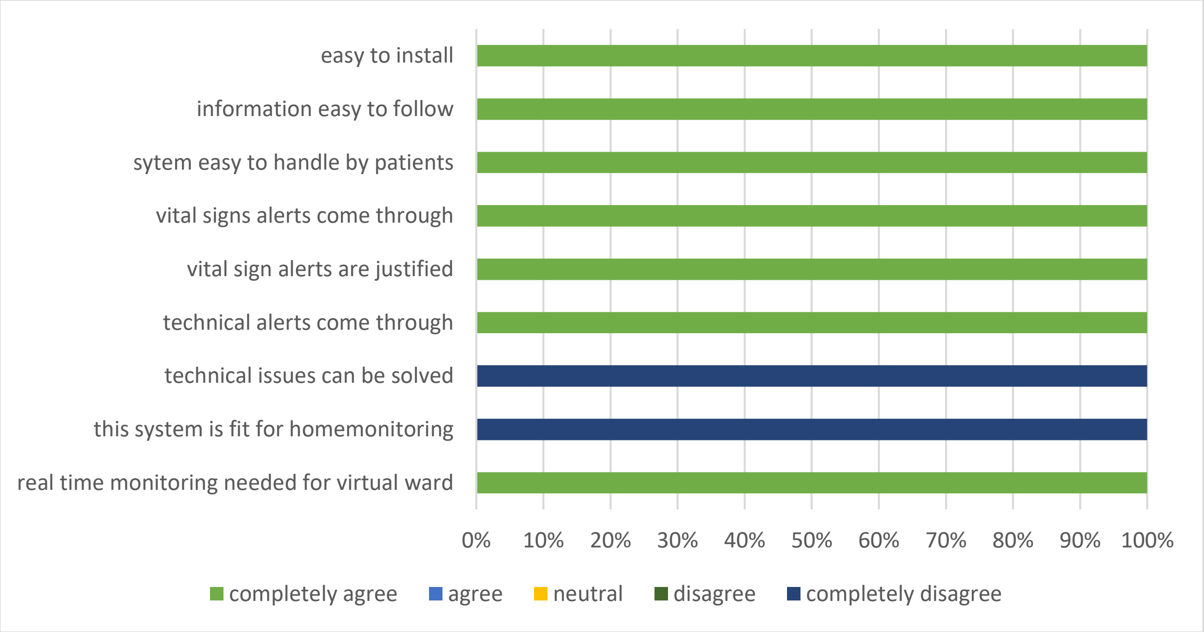

Discussion

In this prospective pilot study, we retrieved perspectives on and experience with telemonitoring in patients visiting the ED with an acute medical condition. In total, about 1 in 7 patients judged it possible/probable to return home with telemonitoring instead of being admitted. The judgement of physicians and nurses on being suitable of returning home instead of being admitted was comparable, however, agreement on this judgement between patients and professionals was only slight-fair. The main reasons not being able to go home were, according to patients, being too seriously ill, the need for treatment that can only be provided in the hospital and the home situation not being safe. The three most mentioned prerequisites were guaranteed admission if necessary, the possibility to contact the treatment team 24/7, preferably by phone, and the presence of someone who could watch over the patient. A subset of these patients tried a telemonitoring system either at home or during admission. The system was feasible for installation in the ED, and was very well-received (easy-to-use, privacy, willing to use it again).

Alternatives for admission in the hospital are currently developed to relieve the strain in the health care system and to provide care that is both well-received by patients and safe. The experience that was gained in chronic care can now be applied to acute care (3, 23). Successful implementation of telemonitoring in (crowded) EDs in patients who may not be stable, is however challenging (15, 24). We believe that before we can start building a new concept, it is important to retrieve the perspectives of patients and acute care professionals. The current study reveals that, although most patients indicated to have no experience with home monitoring, many indicated to be open for the new form of acute care. The reasons for not willing to go home were to be expected, but part of these barriers may be solved in the future, for instance by organizing intravenous medication services. In addition, the positive experience of the telemonitor cohort shows that home monitoring was well-received, which is also seen in other studies (25, 26). The acute care professionals, as well, saw the potential of home monitoring in avoiding admissions. It is important to note that neither patients nor professionals had experience with home monitoring in acute care. Gaining more insight in the opportunities and barriers of telemonitoring in patients and care professionals can be retrieved in qualitative studies and in clinical trials.

Several building blocks for home monitoring as alternative for admission in the hospital have been identified (14): medical care, nursing care, remote monitoring, technology/instruments

and care coordination. Regarding medical care, interestingly, almost all admitted patients received intravenous fluids/medication or oxygen. It is important to retrieve whether this treatment was really necessary as not all medical care can easily and timely be applied in the home situation. It is possible that starting intravenous fluids at the moment of admission is just a routine procedure. Nursing care needs were not retrieved in this study. The presence of someone to watch over the patient was an important prerequisite for home monitoring, but this does not necessarily mean that that someone is a care professional. Interestingly, the patients had many medical instruments at home. This means that it is possible to use these instruments for home monitoring, and/or to build on the skills of the patients. Most of the patients in the telemonitor cohort, however, indicated that they preferred the continuous monitoring of vital signs instead of self-measurements. An additional argument for providing all monitoring material is that this material is validated, and that data can be sent to the treatment team and electronic patient charts. We did not perform analyses into the technology/instruments needed nor into care coordination.

To date, only a few, small randomized controlled studies, in diverse groups of patients, were performed (11-13). These studies were positive – in terms of costs and safety. Interestingly, patients treated in their homes, moved more than hospitalized patients.

It is further important to be able to distinguish between those in need for admission, and those fully fit for further treatment at home. The slight agreement between patients and professionals on this issue that we found is interesting, and probably, at least in part, a lack of communication on telemonitoring between patients and professionals, but this warrants further investigation. Risk stratification scores could be useful for making this distinction (12).

In our study, it was not possible to assess all generated alerts as no corresponding measurements were made in many cases. In addition, patients were, for safety reasons, selected on basis of the absence of need for continuous monitoring and, therefore, few aberrant vital signs were recorded by the patients (self-measurements) or professionals. It was clear, however, that the temperature measurements too often yielded a low body temperature. The main explanation for these incorrect alerts was because of displaced temperature leads. In contrast, in admitted patients, the respiratory rate was often found elevated by the telemonitoring system, while the nurses had noted a respiratory rate of 14. It is known that it is very difficult to measure respiratory rates in clinical practice (27). For future

implementation of telemonitoring in acute care, measurements should be robust, and the most important vital signs should be selected.

Additional limitations of the study are that we had to adapt questionnaires as no relevant validated questionnaires applied to this study (15, 24). More in-depth information on the perspectives of patients and professionals can be retrieved in qualitative studies. In addition, we performed the study during office-hours for practical reasons. It is possible that the proportion of patients willing and able to go home with home monitoring in evenings, nights and weekend differs from that during office hours. However, in a recent, similar study that included the evening, comparable proportions were found (16). A limitation is that patients nor professionals were familiar with the concept of telemonitoring at home. It is therefore possible that they underestimated the potential of telemonitoring.

In conclusion, home monitoring was seen as alternative for admission in a substantial proportion of medical ED patients. Barriers for home monitoring indicated by patients were related to disease severity, need for treatment considered to warrant admission and to a lack of someone to watch over the patient, while a guaranteed admission and 24/7 possibility to contact the treatment team were prerequisites for home monitoring. Although patients had many medical instruments at home, most monitored patients preferred the continuous automated measurements. The agreement in assessment of being fully fit for home monitoring between patients and professionals was low. The telemonitor system tested was feasible for use in the ED, well-received and perceived as comfortable and the concept seems to have potential in reducing the pressure on the health care system.

Funding: none

References

1. Chauhan U, McAlister FA. Comparison of Mortality and Hospital Readmissions Among Patients Receiving Virtual Ward Transitional Care vs Usual Postdischarge Care: A Systematic Review and Meta-analysis. JAMA Netw Open. 2022;5(6):e2219113.
2. Masotta V, Dante A, Caponnetto V, Marcotullio A, Ferraiuolo F, Bertocchi L, et al. Telehealth care and remote monitoring strategies in heart failure patients: A systematic review and meta-analysis. Heart Lung. 2024;64:149-67.
3. Gaillard G, Russinoff I. Hospital at home: A change in the course of care. J Am Assoc Nurse Pract. 2023;35(3):179-82.
4. Shepperd S, Doll H, Angus RM, Clarke MJ, Iliffe S, Kalra L, et al. Admission avoidance hospital at home. Cochrane Database Syst Rev. 2008(4):CD007491.
5. van Goor HMR, Breteler MJM, van Loon K, de Hond TAP, Reitsma JB, Zwart DLM, et al. Remote Hospital Care for Recovering COVID-19 Patients Using Telemedicine: A Randomised Controlled Trial. J Clin Med. 2021;10(24).
6. Chaytee J, Dinh A, D'Anglejan E, Bouchand F, Jaffal K, Duran C, et al. Digital health for remote home monitoring of patients with COVID-19 requiring oxygen: a cohort study and literature review. Front Med (Lausanne). 2023;10:1255798.
7. Fried S, Bar-Shai A, Frydman S, Freund O. Transition of care interventions to manage severe COVID-19 in the ambulatory setting: a systematic review. Intern Emerg Med. 2024;19(3):765-75.
8. Ganjali R, Jajroudi M, Kheirdoust A, Darroudi A, Alnattah A. Telemedicine solutions for clinical care delivery during COVID-19 pandemic: A scoping review. Front Public Health. 2022;10:937207.
9. Patel R, Thornton-Swan TD, Armitage LC, Vollam S, Tarassenko L, Lasserson DS, et al. Remote Vital Sign Monitoring in Admission Avoidance Hospital at Home: A Systematic Review. J Am Med Dir Assoc. 2024;25(8):105080.
10. Varney J, Weiland TJ, Jelinek G. Efficacy of hospital in the home services providing care for patients admitted from emergency departments: an integrative review. Int J Evid Based Healthc. 2014;12(2):128-41.
11. Levine DM, Ouchi K, Blanchfield B, Diamond K, Licurse A, Pu CT, et al. Hospital-Level Care at Home for Acutely Ill Adults: a Pilot Randomized Controlled Trial. J Gen Intern Med. 2018;33(5):729-36.
12. Levine DM, Ouchi K, Blanchfield B, Saenz A, Burke K, Paz M, et al. Hospital-Level Care at Home for Acutely Ill Adults: A Randomized Controlled Trial. Ann Intern Med. 2020;172(2):77-85.
13. Levine DM, Paz M, Burke K, Beaumont R, Boxer RB, Morris CA, et al. Remote vs In-home Physician Visits for Hospital-Level Care at Home: A Randomized Clinical Trial. JAMA Netw Open. 2022;5(8):e2229067.
14. van Goor HMR, de Hond TAP, van Loon K, Breteler MJM, Kalkman CJ, Kaasjager KAH. Designing a Virtual Hospital-at-Home Intervention for Patients with Infectious Diseases: A Data-Driven Approach. J Clin Med. 2024;13(4).
15. Leff B, DeCherrie LV, Montalto M, Levine DM. A research agenda for hospital at home. J Am Geriatr Soc. 2022;70(4):1060-9.
16. Weijers J, Prins MLM, van Dam D, AcuteCare@Home Study G, van Nieuwkoop C, Alsma J, et al. Patients' Perspectives and Feasibility of Home Monitoring in Acute Care: The AcuteCare@Home Flash Mob Study. Telemed J E Health. 2024.
17. Anonymous. [Pharos Quickscan digital skills of your patient]. Website: https://wwwpharosnl/kennisbank/quickscan-digitale-vaardigheden-van-uw-patienten/ (accessed 26-08-2024).
18. Ensink CJ, Keijsers NLW, Groen BE. Translation and validation of the System Usability Scale to a Dutch version: D-SUS. Disabil Rehabil. 2024;46(2):395-400.

19. van der Wulp I, van Baar ME, Schrijvers AJ. Reliability and validity of the Manchester Triage System in a general emergency department patient population in the Netherlands: results of a simulation study. Emerg Med J. 2008;25(7):431-4.
20. Subbe CP, Kruger M, Rutherford P, Gemmel L. Validation of a modified Early Warning Score in medical admissions. QJM. 2001;94(10):521-6.
21. WHO. ICD-10 classification of diseases. Website: https://wwwwhoint/classifications/classification-of-diseases.
22. Landis JR, Koch GG. An application of hierarchical kappa-type statistics in the assessment of majority agreement among multiple observers. Biometrics. 1977;33(2):363-74.
23. Taylor ML, Thomas EE, Snoswell CL, Smith AC, Caffery LJ. Does remote patient monitoring reduce acute care use? A systematic review. BMJ Open. 2021;11(3):e040232.
24. Pannunzio V, Morales Ornelas HC, Gurung P, van Kooten R, Snelders D, van Os H, et al. Patient and Staff Experience of Remote Patient Monitoring-What to Measure and How: Systematic Review. J Med Internet Res. 2024;26:e48463.
25. Haddad TC, Maita KC, Inselman JW, Avila FR, Torres-Guzman RA, Coffey JD, et al. Patient Satisfaction With a Multisite, Multiregional Remote Patient Monitoring Program for Acute and Chronic Condition Management: Survey-Based Analysis. J Med Internet Res. 2023;25:e44528.
26. Hayden EM, Nentwich LM, Jung OS, Zheng H, White BA. Patient Perceptions of Emergency Department Observation Care at Home. Telemed J E Health. 2024;30(7):1874-9.
27. Semler MW, Stover DG, Copland AP, Hong G, Johnson MJ, Kriss MS, et al. Flash mob research: a single-day, multicenter, resident-directed study of respiratory rate. Chest. 2013;143(6):1740-4.

supplemental tables

Click here to access/download
Supporting Information
supplemental data TM acute care.docx

dataset

Click here to access/download
Supporting Information
TM acute care_1.sav
