# Supplementary material for: Telemonitoring starting in the emergency department as an alternative to acute hospital admission: A prospective pilot study focusing on patient preferences and first experience
Source: PLOS Digit Health. 2025 Jul 31;4(7):e0000962. doi: 10.1371/journal.pdig.0000962 (PMC12312925; doi:10.1371/journal.pdig.0000962)
Supplement: Attachment — Submitted filename: rebuttal plos digital health.docx [file pdig.0000962.s014.docx]

PDIG-D-25-00071

Telemonitoring at home as alternative for admission in acute care. A prospective pilot study focusing on patient perspectives and first experiences

PLOS Digital Health

Dear Dr. Stassen,

Thank you for submitting your manuscript to PLOS Digital Health. After careful consideration, we feel that it has merit but does not fully meet PLOS Digital Health's publication criteria as it currently stands. Therefore, we invite you to submit a revised version of the manuscript that addresses the points raised during the review process.

Please submit your revised manuscript within 60 days Apr 26 2025 11:59PM. If you will need more time than this to complete your revisions, please reply to this message or contact the journal office at [digitalhealth@plos.org](mailto:digitalhealth@plos.org). When you're ready to submit your revision, log on to <https://www.editorialmanager.com/pdig/> and select the 'Submissions Needing Revision' folder to locate your manuscript file.

Please include the following items when submitting your revised manuscript:

* A rebuttal letter that responds to each point raised by the editor and reviewer(s). You should upload this letter as a separate file labeled 'Response to Reviewers'. This file does not need to include responses to any formatting updates and technical items listed in the 'Journal Requirements' section below.

* A marked-up copy of your manuscript that highlights changes made to the original version. You should upload this as a separate file labeled 'Revised Manuscript with Track Changes'.

* An unmarked version of your revised paper without tracked changes. You should upload this as a separate file labeled 'Manuscript'.

If you would like to make changes to your financial disclosure, competing interests statement, or data availability statement, please make these updates within the submission form at the time of resubmission. Guidelines for resubmitting your figure files are available below the reviewer comments at the end of this letter.

We look forward to receiving your revised manuscript.

Kind regards,

Haleh Ayatollahi

Section Editor

PLOS Digital Health

Haleh Ayatollahi

Section Editor

PLOS Digital Health

Leo Anthony Celi

Editor-in-Chief

PLOS Digital Health

orcid.org/0000-0001-6712-6626

**Dear editor and dear reviewers,**

**Thank you for your thorough review of our manuscript.**

**We have carefully read your remarks and adjusted the manuscript accordingly.**

**We think that your input has led to an improved manuscript for which we are grateful.**

**Please find our responses below.**

**We will refer to the numbers of the lines in the manuscript with track changes to make the changes as clear as possible.**

**.**

**Journal Requirements:**

1. We noticed that you used “data not shown” in the manuscript. We do not allow these references, as the PLOS data access policy requires that all data be either published with the manuscript or made available in a publicly accessible database. Please amend the supplementary material to include the referenced data or remove the references.

We removed these phrases from the results section and added the exact agreement analyses to the supplemental tables.

**Additional Editor Comments (if provided):**

[Note: HTML markup is below. Please do not edit.]

**Reviewers' Comments:**

Reviewer's Responses to Questions

**Comments to the Author**

1. Does this manuscript meet PLOS Digital Health’s [publication criteria](https://eur01.safelinks.protection.outlook.com/?url=https%3A%2F%2Ftrack.editorialmanager.com%2FCL0%2Fhttps%3A%252F%252Fjournals.plos.org%252Fdigitalhealth%252Fs%252Fjournal-information%2523loc-criteria-for-publication%2F1%2F010f01953f7f71ee-86ec1e93-8409-4c81-b680-0220996cb733-000000%2FAwJqbUnttKk60hdcRInFVOOH6MQsxhFd9eQ4yCGK9k0%3D199&data=05%7C02%7Cp.stassen%40mumc.nl%7C9a543cec7bad46996ed608dd55f65b4c%7C08273b66221040cd8b205cf6cddc279a%7C0%7C0%7C638761239067359437%7CUnknown%7CTWFpbGZsb3d8eyJFbXB0eU1hcGkiOnRydWUsIlYiOiIwLjAuMDAwMCIsIlAiOiJXaW4zMiIsIkFOIjoiTWFpbCIsIldUIjoyfQ%3D%3D%7C0%7C%7C%7C&sdata=9C7dp7Ac0q%2F65V5anispsmh1hdsGxhDzJGlvuUIVw1A%3D&reserved=0)? Is the manuscript technically sound, and do the data support the conclusions? The manuscript must describe methodologically and ethically rigorous research with conclusions that are appropriately drawn based on the data presented.

Reviewer #1: Partly

Reviewer #2: Yes

2. Has the statistical analysis been performed appropriately and rigorously?

Reviewer #1: Yes

Reviewer #2: Yes

3. Have the authors made all data underlying the findings in their manuscript fully available (please refer to the Data Availability Statement at the start of the manuscript PDF file)?

The [PLOS Data policy](https://eur01.safelinks.protection.outlook.com/?url=https%3A%2F%2Ftrack.editorialmanager.com%2FCL0%2Fhttps%3A%252F%252Fjournals.plos.org%252Fdigitalhealth%252Fs%252Fdata-availability%2F1%2F010f01953f7f71ee-86ec1e93-8409-4c81-b680-0220996cb733-000000%2FNEP0IFSCXHbaZ2uoGZWkHOpyUvPjjH9bPwIAqKrhgOo%3D199&data=05%7C02%7Cp.stassen%40mumc.nl%7C9a543cec7bad46996ed608dd55f65b4c%7C08273b66221040cd8b205cf6cddc279a%7C0%7C0%7C638761239067376639%7CUnknown%7CTWFpbGZsb3d8eyJFbXB0eU1hcGkiOnRydWUsIlYiOiIwLjAuMDAwMCIsIlAiOiJXaW4zMiIsIkFOIjoiTWFpbCIsIldUIjoyfQ%3D%3D%7C0%7C%7C%7C&sdata=RuMgT3Fwu7beYF4fv3XzUMBrVhomBGXAo9Re7Pd5bu8%3D&reserved=0) requires authors to make all data underlying the findings described in their manuscript fully available without restriction, with rare exception. The data should be provided as part of the manuscript or its supporting information, or deposited to a public repository. For example, in addition to summary statistics, the data points behind means, medians and variance measures should be available. If there are restrictions on publicly sharing data—e.g. participant privacy or use of data from a third party—those must be specified.

Reviewer #1: Yes

Reviewer #2: Yes

4. Is the manuscript presented in an intelligible fashion and written in standard English?

PLOS Digital Health does not copyedit accepted manuscripts, so the language in submitted articles must be clear, correct, and unambiguous. Any typographical or grammatical errors should be corrected at revision, so please note any specific errors here.

Reviewer #1: No

Reviewer #2: Yes

5. Review Comments to the Author

Please use the space provided to explain your answers to the questions above. You may also include additional comments for the author, including concerns about dual publication, research ethics, or publication ethics. (Please upload your review as an attachment if it exceeds 20,000 characters)

Reviewer #1: Dear authors,
The aim of the study is good. It needs to be supported in the introduction. The introduction part is simple and careless. Also, some undisclosed data is quite important and necessary. I uploaded the file. The arrangements are presented on the file.

Thank you for your thorough and constructive review and your interesting remarks. We hope that we understood your remarks correctly, because due to brevity and placement of the boxes we were not always sure what you meant to say. We tried to adjust the manuscript according to these remarks as concise as possible and as detailed below.

You made three remarks on the Introduction section.

The first being on providing a reference to the preferences of patients to design new forms of care.

We added a reference to support our statement. (line 54)

The second remark is on a lack of clarity on the telemonitoring concept.

We added a sentence to clarify this concept. In this stage of the manuscript, we refer to a general concept, where health care is provided from a distance. We changed the sentence to make this more clear.

.. By monitoring specific aspects of health and disease, patients can manage their health or disease within their own environment…(added: )“ in close collaboration with and supervision by their treatment team, but without actual physical contact”. (lines 55-57)

In the following lines we focused on chronic care, and then on acute care.

Your third remark was on “continuous telemonitoring started in the ED being only by interpreted by doctors” .

We aimed to investigate preferences and experiences of patients regarding telemonitoring. We therefore asked ED patients regarding their preferences/prerequisites for homemonitoring, and performed the current study during which ED patients were asked to try telemonitoring. We further asked doctors to share their experience with telemonitoring. Only doctors were involved in the actual monitoring of the patients.

We agree with you, however, that it would be interesting to involve patients and other care professionals in the interpretation of the data, and we added a remark on this issue to the limitations in the Discussion.

“Gaining more insight in the opportunities and barriers of telemonitoring in patients and care professionals can be retrieved in qualitative studies and in clinical trials, (added:),… which involve patients in the setup and interpretation of the results, ….” (lines 356-357)

In the methods section, you made a remark on “the ED patient” being very broad.

ED patients in general constitue indeed a very broad/diverse group , but we included patients treated with internal medicine problems only (as stated in the inclusion criteria, lines 104-107), and in Table 1, their characteristics are shown. We are unsure what more details should be provided.

You further ask on literature to support our inclusion criteria or do you mean literature on the questionnaires?

The literature on which the questionnaire is added in the section on Data collection.

We are not sure how to respond to the remark if you meant to comment on the inclusion criteria. We included all patients with internistic problems within the study period who were willing and able to answer our questionnaire. Although we always asked informed consent, sometimes, it was deemed not possible to ask permission to avoid putting too much strain on already stressed patients/caretakers (lines 104-107). As far as we know, there is no standard definition on how to include ED patients in prospective studies.

You placed a remark on the sample of ED patients, in part being sent home. These patient being sent home were treated as outpatients.

There were indeed patients who were treated as outpatients after their ED visit. About 60% of the ED patients who are assessed and treated by internists are usually admitted, the rest is not. In our opinion, these are still ED patients.

The aim of our study was to retrieve preferences in patients in the acute setting of an ED. These ED patients all had an acute, unplanned medical problem and experienced an amount of stress. We aimed to investigate how these patients reacted to the proposition of going home with telemonitoring instead of being admitted, in the future. In other words: they were presented with the concept of homemonitoring, in the context of an unplanned and probably stressful ED visit. This context differs substantially from the chronic setting, where there is time to adjust to new technology and to make decisions after careful consideration. This difference in context is pointed out in the Introduction (first/second paragraph). We conclude, however, that we have not been clear enough on this topic and therefore changed the text on the aims of the study to make these more clear.

We reformulated this in the Title, Introduction and Methods and made it more clear that this study focuses on continued care after ED visit (either as an outpatient or as an admitted patient).

Examples of our revision:

Title: Telemonitoring at home for provision of continued care in the acute care setting

Line 81 (Introduction): added to the last paragraph (line 81): …”continued care starting in the ED”

line 95-96 methods:

..added: “to test telemonitoring as a possible form of continued care.”

You further asked for more information on the consent of the telemonitored patients.

We added more details on this topic:

They were asked additional (added:) informed consent to wear a telemonitoring system for at least 24 hours ……(added: ) and to answer questionnaires on their experience with the telemonitor system. (line 110-112)

You further asked how we dealt with failures/alarms.

As soon as patients returned home, we checked the signal and we contacted patients if necessary regarding the connections. If there were technical alerts later on, we contacted the patient and tried to solve them. We experienced, however, that technical errors could not be handled easily. That was a reason to stop the monitoring session, as depicted in Figure 3. Patients were selected only, when not in need for monitoring as stated in line 112-117.

If vital signs alerts were raised, we contacted the patients to check on them.We finished the monitoring after at least 24 hours or when the patient wished to stop.

“If the vital signs or alerts prompted further investigation or action, two of the investigators (experienced acute internists) contacted the treatment team (admitted patients) or patients (at home)” (see lines 132-134).

You made a remark on the flow chart (missed and revised?).

We are not sure whether we understood your remark correctly, but we rephrased our inclusion criteria in such a way that the phrasing regarding the exclusion criteria is similar to that used in the methods section.

Our exclusion criteria were: ED revisit (patients could be included only once during the study period), being too severely ill or confused to answer the questions in a reliable way without putting too much strain on patient or family (i.e. no consent possible), and a language barrier.

Now rephrased to:

Exclusion criteria were: refusal of consent, being too severely ill or confused to answer the questions in a reliable way without putting too much strain on patient or family (no consent possible), ED revisit (patients could be included only once during the study period), and a language barrier. In addition, we anticipated that some patients would be missed because due to crowding it would not be possible to ask informed consent. Lines 104-107

Regarding your remark on the lay-out of Table 1:

this table was adapted.

You ask whether patients who had experience with telemonitoring were excluded.

They were not excluded, and there were only 7 who had experience. Notably, none had experience in acute care. We added this to line 231:

..”none in the setting of acute care”.

You made a remark on “data not shown” in the results section regarding agreement between nurses and patients.

We added a table (3B) to the supplemental files.

Reviewer #2: This is a very interesting and timely paper given the exponential increase in home monitoring technologies being implemented across the globe. While the authors recognize the lack of research in this area, they overstate the safety aspects of this pilot. The potential application of the findings is quite limited given the small sample size and limited information gathered in this study. It still remains unclear whether telemonitoring should be used as an alternative for ED admission.

Thank you for your review and for your constructive feedback. Please find the changes made according to your remarks below.

We agree with the reviewer that we cannot conclude on safety. This was indeed not the aim of this study. Instead, this was a pilot study during which we investigated the experience of patients with telemonitoring, while they were not in need of that continuous (tele) monitoring. See lines 111-117 in the methods). We agree on the small sample size.

We adjusted the manuscript to clarify the context of our results

Examples of our adjustments are: adjustment of title

Methods:

Line 117-119: Addition of the sentence:

We made this conservative selection of patients because we aimed to retrieve experiences of patients with the telemonitoring concept; assessment of safety was not within the scope of this study.

Discussion

Line 373: Evaluation of safety was not within the scope of our study because…...

Lines 382-383: We added ….For future implementation of telemonitoring in acute care, (added):…safety should be established, measurements should be robust, and the most important vital signs should be selected.

Of most concern is the fact that the telemonitoring cohort that returned to hospital received more initial treatment than the ED cohort. (See Table 1, Initial treatment after admission, page 10). This sees to indicate that they were much sicker and required more care and attention than the ED cohort. This needs to be more clearly addressed as this program may not in fact be as useful a strategy as the authors may have thought.

Our telemonitor cohort included ED patients who were subsequently either admitted or non-admitted patients. None of the patients were in need of continuous monitoring and, as for those sent home, in need of admission. (lines 111-117, methods).

We aimed to test the feasibility of starting homemonitoring in the setting of an ED, and to retrieve the experience of the patients with such a system. It is too early to conclude that homemonitoring is a safe alternative for admission. Because admitted patients were part of the telemonitor cohort, there were some ill seriously ill patients in the cohort, and differences were to be expected. By including admitted patients, we aimed to retrieve some comparison with vital signs measured in the hospital and to find out whether alerts were indeed generated by the monitorsystem.

We added some clarifying remarks to this topic in the Methods, in the Discussion (see our response to your earlier comment), and in the Conclusion

We added this to the methods section: (lines 127-128)

All patients received care as usual with the exception of daily phone calls on their experiences.

Line 117 (methods): added: …assessment of safety was not within the scope of this study.

Lines 410-412 (conclusion): Added: No conclusions on safety can be drawn, …but the telemonitor system tested was feasible for use in the ED, well-received and perceived as comfortable and the concept seems to have potential in reducing the pressure on the health care system..

In the title they say the study focuses on patient perspectives and first experiences. However there is a substantial lack of qualitative data to describe in-depth these perspectives and experiences. The authors are encouraged to rephrase the title and add their methodology for clarity.

We performed a first quantitative exploration on perspectives of patients. We used (adapted) questionnaires to retrieve these perspectives. We agree with the reviewer that this study was not a qualitative study.

We adjusted the title and added some clarification in the Discussion.

In the Discussion, we state: More in-depth information on the perspectives of patients and professionals can be retrieved in qualitative studies, which may build upon the preferences and barriers identified in this study”” (lines 387-388)

The inclusion exclusion criteria are unclear. For example – what does ‘severely ill’ mean? Most individuals receiving care at the ED could be considered ‘severely ill’. So more clarity is needed.

The majority of patients were asked for participation. However, sometimes, the research team deemed it inappropriate to ask informed consent to either patient or family. If patients were severely ill, most of the times, family members answered our questions. In total, 6 patients were excluded because the patient was too severely ill, eg. because the patient had to be intubated or had to receive vasopressive medication because of septic shock. In these cases, raising questions on homemonitoring to either patient or family member was deemed not appropriate/possible.

We made the following adjustment to the inclusioncriteria (lines 104-107)

…being too severely ill or confused to answer the questions in a reliable way without putting too much strain on both patient and family (no consent possible),

The rationale for the groups was unclear. (See lines 103-110, page 4, the second paragraph under patients). Please add more detail.

In this study, we had two objectives:

1) to retrieve preferences of patients on homemonitoring. All included patients were adults with problems treated by internists.

2) to test homemonitoring in selected patients; at least patients had to live within a 15 km radius from the hospital (when discharged home) or not being in need of continuous monitoring (admitted group).

We clarified the inclusion criteria (lines 99-117).

And we added a rationale for this selection (lines 117-119)

We made this conservative selection of patients because we aimed to retrieve experiences of patients with the telemonitoring concept; assessment of safety was not within the scope of this study.

On the tables, please specify N and % at the top of the specific columns. For example, as Table 1 reads now, column 1is noted to show N (%) while this is actually the case for columns 2 and 3. Column 1 actually shows the Patient characteristic.

We changed the lay-out across the manuscript to make this more clear.

Table 4 requires more detail as it simply states Patient: capable. Would be helpful to specify capable of what? The narrative notes three domains. In table 3 must they be capable of all three domains, or one or more of the three? It is hard to follow the details of what was done here.

We agree that this is not completely clear. We added extra information in a subscript to make this more clear.

Bar charts are hard to read when printed in black and white. Suggest the authors recolour these using black and white patterns with outlined bars to ensure readers can follow. Also, capitalization is needed in the headings on Figures 2A, 2B, and 3.

We have reformatted the figures according to the requirements of Plos Digital Health.

Following the instructions below (under the review), we resubmitted the figure. Hopefully it will be understandable to the readers.

Further details on the specifics of the monitoring are needed. How did participants learn how to use them. Were they self-monitored or monitored by a care partner? Was a specific type of technology used? If so, please specify this. Not enough information is available for a reader who may be interested to reproduce or expand on this work.

The system we used is very robust and does not require actions from the patients. We added a sentence to the methods section to make this more clear and to allow for reproduction of our study.

No actions were required from the patients. (line 129-130)

6. PLOS authors have the option to publish the peer review history of their article ([what does this mean?](https://eur01.safelinks.protection.outlook.com/?url=https%3A%2F%2Ftrack.editorialmanager.com%2FCL0%2Fhttps%3A%252F%252Fjournals.plos.org%252Fdigitalhealth%252Fs%252Feditorial-and-peer-review-process%2523loc-peer-review-history%2F1%2F010f01953f7f71ee-86ec1e93-8409-4c81-b680-0220996cb733-000000%2FRn6B0QZehkDlbMQ-N9Ue2VF2RG8z_M7k1RLVT3H1T0s%3D199&data=05%7C02%7Cp.stassen%40mumc.nl%7C9a543cec7bad46996ed608dd55f65b4c%7C08273b66221040cd8b205cf6cddc279a%7C0%7C0%7C638761239067388876%7CUnknown%7CTWFpbGZsb3d8eyJFbXB0eU1hcGkiOnRydWUsIlYiOiIwLjAuMDAwMCIsIlAiOiJXaW4zMiIsIkFOIjoiTWFpbCIsIldUIjoyfQ%3D%3D%7C0%7C%7C%7C&sdata=%2BNksYcqFq%2F3qYPTJ1jqGEl2rwgxk4is0jB4T3tUwrfA%3D&reserved=0)). If published, this will include your full peer review and any attached files.


**Do you want your identity to be public for this peer review?** If you choose “no”, your identity will remain anonymous but your review may still be made public.


For information about this choice, including consent withdrawal, please see our [Privacy Policy](https://eur01.safelinks.protection.outlook.com/?url=https%3A%2F%2Ftrack.editorialmanager.com%2FCL0%2Fhttps%3A%252F%252Fwww.plos.org%252Fprivacy-policy%2F1%2F010f01953f7f71ee-86ec1e93-8409-4c81-b680-0220996cb733-000000%2FeuP2_fFJEFer_3cPXz86LPh6e-_4PeCkmpWvKKI9TS8%3D199&data=05%7C02%7Cp.stassen%40mumc.nl%7C9a543cec7bad46996ed608dd55f65b4c%7C08273b66221040cd8b205cf6cddc279a%7C0%7C0%7C638761239067401610%7CUnknown%7CTWFpbGZsb3d8eyJFbXB0eU1hcGkiOnRydWUsIlYiOiIwLjAuMDAwMCIsIlAiOiJXaW4zMiIsIkFOIjoiTWFpbCIsIldUIjoyfQ%3D%3D%7C0%7C%7C%7C&sdata=2KQGHlsVdCV%2F%2BZddDrPLhwG9WUJSCHi4LXjG3MGT358%3D&reserved=0).

Reviewer #1: No

Reviewer #2: No

[NOTE: If reviewer comments were submitted as an attachment file, they will be attached to this email and accessible via the submission site. Please log into your account, locate the manuscript record, and check for the action link "View Attachments". If this link does not appear, there are no attachment files.]

**Figure resubmission:**

While revising your submission, please upload your figure files to the Preflight Analysis and Conversion Engine (PACE) digital diagnostic tool, <https://pacev2.apexcovantage.com/>. PACE helps ensure that figures meet PLOS requirements. To use PACE, you must first register as a user. Registration is free. Then, login and navigate to the UPLOAD tab, where you will find detailed instructions on how to use the tool. If you encounter any issues or have any questions when using PACE, please email PLOS at [figures@plos.org](mailto:figures@plos.org). Please note that Supporting Information files do not need this step. If there are other versions of figure files still present in your submission file inventory at resubmission, please replace them with the PACE-processed versions.

**Reproducibility:**

To enhance the reproducibility of your results, we recommend that authors of applicable studies deposit laboratory protocols in protocols.io, where a protocol can be assigned its own identifier (DOI) such that it can be cited independently in the future. Additionally, PLOS ONE offers an option to publish peer-reviewed clinical study protocols. Read more information on sharing protocols at <https://plos.org/protocols?utm_medium=editorial-email&utm_source=authorletters&utm_campaign=protocols>
