# Supplementary material for: Telemonitoring starting in the emergency department as an alternative to acute hospital admission: A prospective pilot study focusing on patient preferences and first experience
Source: PLOS Digit Health. 2025 Jul 31;4(7):e0000962. doi: 10.1371/journal.pdig.0000962 (PMC12312925; doi:10.1371/journal.pdig.0000962)
Supplement: Attachment — Submitted filename: PLOS Digital Health Review PDIG-D-25-00071R1.docx [file pdig.0000962.s013.docx]

**PLOS DIGITAL HEALTH MANUSCRIPT REVIEW**

**Manuscript Number: PDIG-D-25-00071R1**

**Title: Telemonitoring at home for provision of continued care in the acute care**

**setting. A prospective pilot study focusing on first experiences of patients**

**Comments:**

1. The original submission focused on the use of telemonitoring as an alternative to hospital admission and emergency room admissions. In response to the first submission reviewer's comments the authors have now changed the title to one which frankly is less clear that of telemonitoring as a prelude for provision of subsequent acute care. It would be best to stick to what was originally decided as the focus of the study rather than *post hoc* shifting titles to a review or comment which then is really not aimed at what the study was all about. I suggest the authors think this through carefully and come up with a crisp title
2. Similarly, the same follows through in the abstract. The abstract states that telemonitoring may be used to reduce admissions to the ER and that they performed the pilot study to gain the perspectives and experiences of patients with telemonitoring - yet that sentence doesn't have a completion. Did the study aim at looking at reduction of ED admissions, or the experience in the ED or subsequent care??? The second sentence in the introduction of abstract needs to be fixed.
3. In the Results section of the abstract. This sentence needs to be clarified. “For professionals, these proportions were lower, and agreement with patients was slight-fair. ????
4. In the conclusion of the abstract. These sentences need to be clarified. “Prerequisites were guaranteed admission and 24/7 contact with the treatment team, while barriers were related to disease severity and lack of someone to watch over the patient. Telemonitoring can be used in acute care as alternative for admissions if prerequisites are met and. barriers removed. “ Barriers ??? – not clear
5. Entry criteria are still not clear. Was it all commers?
6. Need a breakdown of what were the disease – more granularity that what was provided , e.g. for CV there may be differences for a patient with HTN or chroinic angina vs one with stuttering angina or unstable angina – need this kind of information ( or if unavailable need to comment on tis in the discussion) , the acute conditions being monitored, and/or Diagnosis at the time of admission. Very important.
7. Discussion. Line 349. Several building blocks for home monitoring as alternative for admission in the hospital have been identified (15): Building blocks. – not clear, poor choice of term
8. Discussion Line 405. The agreement in assessment of being fully fit for home

monitoring between patients and professionals was low. The numbers were so low. Need t comment on that. Its hard to distinguish patient vs professional opinion.

**Overall, this is an important study, but the message is getting lost with lack of clarity. Suggest carefully outline the top 3-5 bullet points that you gleaned from the study – put these in the abstract and in the discussion and the paper will be much better.**
