# Supplementary material for: Telemonitoring starting in the emergency department as an alternative to acute hospital admission: A prospective pilot study focusing on patient preferences and first experience
Source: PLOS Digit Health. 2025 Jul 31;4(7):e0000962. doi: 10.1371/journal.pdig.0000962 (PMC12312925; doi:10.1371/journal.pdig.0000962)
Supplement: Attachment — Submitted filename: Response to reviewers TM acute care revision 2.docx [file pdig.0000962.s015.docx]

**PDIG-D-25-00071R1**

**Telemonitoring at home for provision of continued care in the acute care setting. A prospective pilot study focusing on first experiences of patients**

**PLOS Digital Health**

**Dear Dr. Stassen,**

**Thank you for submitting your manuscript to PLOS Digital Health. After careful consideration, we feel that it has merit but does not fully meet PLOS Digital Health's publication criteria as it currently stands. Therefore, we invite you to submit a revised version of the manuscript that addresses the points raised during the review process.**

Dear editor and reviewers,

Thank you for your thorough review and for finding (new) reviewers again.

We adjusted the manuscript according to the comments made, which improved the manuscript in our opinion. Please find our responses to the comments below.

With Kind regards,

on behalf of all authors,

Patricia Stassen

**Reviewer #5:**

**Title: Telemonitoring at home for provision of continued care in the acute care**

**setting. A prospective pilot study focusing on first experiences of patients**

**Comments:**

1. The original submission focused on the use of telemonitoring as an alternative to hospital admission and emergency room admissions. In response to the first submission reviewer's comments the authors have now changed the title to one which frankly is less clear that of telemonitoring as a prelude for provision of subsequent acute care. It would be best to stick to what was originally decided as the focus of the study rather than *post hoc* shifting titles to a review or comment which then is really not aimed at what the study was all about. I suggest the authors think this through carefully and come up with a crisp title.

Response: It is indeed important to chose a concise title. We did not aim to find alternatives to ED admissions, but hospital admissions after ED visit. Our aim was to test the telemonitor concept by collecting perspectives of patients, judgements of professionals and patients on suitabilty for telemonitoring, and to test a monitorsystem in a subset of patients.

We changed the title to

Telemonitoring starting in the emergency department as alternative to acute hospital admission

A prospective pilot study focusing on patient preferences and first experience

2. Similarly, the same follows through in the abstract. The abstract states that telemonitoring may be used to reduce admissions to the ER and that they performed the pilot study to gain the perspectives and experiences of patients with telemonitoring - yet that sentence doesn't have a completion. Did the study aim at looking at reduction of ED admissions, or the experience in the ED or subsequent care??? The second sentence in the introduction of abstract needs to be fixed.

Response: We agree that the aim of the study should be perfectly clear to the reader. We therefore changed the aim in the abstract to:

“We performed a pilot study to investigate the perspectives and experiences of ED patients and care professionals with telemonitoring started in the ED used as potential alternative to acute hospital admission.”

3. In the Results section of the abstract. This sentence needs to be clarified. “For professionals, these proportions were lower, and agreement with patients was slight-fair. ????

These sentences are indeed somewhat too short to be clear to the reader. We therefore changed the paragraph to:

“In total, 11.2% of patients indicated that admission could be avoided using telemonitoring, while another 6.1% thought this might be possible. Professionals judged less patients capable to be sent home with telemonitoring (physicians: 7.2% and 6.1%, resp.; nurses: 10.4% and 4.2%, resp.). Agreement on the capability of patients to be sent home with telemonitoring between patients and professionals was slight-fair.”

4. In the conclusion of the abstract. These sentences need to be clarified. “Prerequisites were guaranteed admission and 24/7 contact with the treatment team, while barriers were related to disease severity and lack of someone to watch over the patient. Telemonitoring can be used in acute care as alternative for admissions if prerequisites are met and. barriers removed. “ Barriers ??? – not clear

We changed the text to make this more clear. In implementation literature, it is indeed common to speak of facilitators and barriers.

In the last sentence, the phrase..”and barriers removed” was deleted, because it is not possible to remove the barrier of disease severity and solving the problem that some patients live alone and have no one to watch over them. Some patients are, and will always be, too severely ill to be sent home.

“Telemonitoring at home was seen as alternative to admission in a substantial proportion of medical ED patients. Facilitators for telemonitoring indicated by patients were guaranteed admission if telemonitoring failed and the possiblilty to contact the treatment team 24/7, while indicated barriers were related to disease severity and lack of someone to watch over the patient. Telemonitoring can be used in acute care as alternative for admissions if facilitators are met.”

5. Entry criteria are still not clear. Was it all commers?

We indeed included all patients who visited the ED with problems handled by internists in a certain timeframe only (ED cohort). We provided inclusion and exclusion criteria.

These patients completed the questionnaire. A subset of these patients were subsequently selected, based on additonal inclusion criteria (such as living near the hospital and extra consent; telemonitor cohort).

We rephrased this section to make this more clear:

“Part of the ED patients were selected to form a second cohort (telemonitor cohort). Patients were eligible for this cohort if they provided additional informed consent to wear a telemonitoring system for at least 24 hours and to answer questionnaires on their experience with the telemonitor system. In addition they had to live within a 15 km radius of the hospital. Furthermore,….”

6. Need a breakdown of what were the disease – more granularity that what was provided , e.g. for CV there may be differences for a patient with HTN or chroinic angina vs one with stuttering angina or unstable angina – need this kind of information ( or if unavailable need to comment on tis in the discussion) , the acute conditions being monitored, and/or Diagnosis at the time of admission. Very important.

We provided ED diagnoses in Table 1, categorized according to ICD-10 diagnoses. We included patients with problems handled by internists only, so no patients with unstable angina were included. In this study, the patients with cardiovascular problems had hypertension (n=3), syncope (n=2) and non-severe heartfailure (n=1).

We added info on more exact diagnoses in the supplementary section (suppl Table 1).

7. Discussion. Line 349. Several building blocks for home monitoring as alternative for admission in the hospital have been identified (15): Building blocks. – not clear, poor choice of term

We chose the words building blocks, because the authors of the study we refer to used this phrasing as well. We however agree with you that the word choice could be better. We changed the sentence to..

“Several components for home monitoring…”

8. Discussion Line 405. The agreement in assessment of being fully fit for home

monitoring between patients and professionals was low. The numbers were so low. Need t comment on that. Its hard to distinguish patient vs professional opinion.

We agree that the number of judgements on avoidance of admission (table 3) is small. We added this limitation to the discussion/limitations.

“This resulted in a relatively small study population, possibly resulting in a lack of power regarding analyses of, for instance, judgement of avoidance of admission.”

Overall, this is an important study, but the message is getting lost with lack of clarity. Suggest carefully outline the top 3-5 bullet points that you gleaned from the study – put these in the abstract and in the discussion and the paper will be much better.

We deleted some of the data or moved these to the supplementary data to make the study less “data heavy” (mentioned by one of the other reviewers). We hope the manuscript is more clear now.

Thank you for your constructive feedback.

**Reviewer #3:**

**This is a timely and well-motivated pilot study exploring an emerging area—telemonitoring in acute care. The revised manuscript has addressed many of the initial reviewer concerns thoughtfully and effectively. It is significantly improved in framing its objectives, clarifying methods, and reinforcing its identity as a feasibility and experience-based study rather than a safety evaluation. However, several areas would still benefit from further clarification and precision to strengthen reproducibility and overall interpretability:**

Thank you for your review and compliments. Please find our detailed responses to your comments below.

**• While the authors now clarify that safety was not assessed, statements like “telemonitoring can be used in acute care as alternative for admissions if prerequisites are met and barriers removed” might still feel conclusive. Suggest rephrasing as “telemonitoring may serve as a potential alternative…” in the abstract and conclusion to avoid overinterpretation.**

We indeed performed a pilot study to test the concept of telemonitoring in the setting of acute care (questionnaire and experience with a monitoring device), and we did not aim to test safety. We agree that we cannot conclude on safety.

To make this absolutely clear, we changed the text according to your suggestion.

“Telemonitoring in acute care may serve as potential alternative for admissions if prerequisites are met.”

**• Reviewer #2’s concern about the telemonitoring setup remains only partially addressed. Key details—such as the specific type, name, or model of the device used (if applicable)—are still lacking. This information is important to ensure reproducibility and allow others to build on this work.**
We aimed to test the concept of telemonitoring consisting of a device that warrants no further action from the patient. We added a reference.

**• The concept of being “capable” (Table 4) is still not fully clear. Since this refers to three domains (physical, cognitive, support), it would be helpful to rephrase as “fully capable in all three domains with an asterisk” consistently across tables and text. The asterisk can be used explain the 3 domains in the table’s caption.**

We changed the table and text (methods/outcome measures)**.

• Table 1 reports separately on patients being community-dwelling, with a roommate, and having an informal caregiver. However, the relationship between these categories remains unclear. Is there overlap between those who live with a roommate and those who have an informal caregiver? This is important because, if I interpret “roommate” and “informal caregiver” as distinct subsets of the community-dwelling group, the reported percentages do not logically add up. Additionally, it appears that the one non–community-dwelling patient was omitted from Table 1—please clarify whether this was intentional.**

Patients were asked whether they lived alone or not. The subsequent question was: Is there someone who can help you (partner/informal caretaker). Sometimes, the roommate/partner was not able to provide support, and sometimes, a patient lived alone but had a friendly neighbour or familymember living nearby who could provide support.

See supplemental file 1 (preference questionnaire).

Your second remark is on the denominator we used regarding the living situation? If so, this is a sharp observation; indeed, if someone is not community dwelling, there will always be someone to take care of the patient. We checked the data, and corrected the table.

**• Table 3, clearer legends are needed to explain how “Home without telemonitoring” fits within the other categories (i.e., are they excluded from kappa analysis?).**
Indeed, regarding the agreement on possible avoidance of admission by using telemonitoring, agreement was calculated in patients who were admitted to the hospital only. This was added (more clearly) to the methods/analysis and to the table.

**• The reviewer 2 comments on Table 4 was not fully addressed. Clarify that physicians rated capability based on the combined three domains, and also, specify whether being classified as “not fully capable” means the patient failed in any one of the domains, or in all three.**
We added this to the legend: ..”not fully: not capable in at least one domain..”

**• It is mentioned that 12 patients went home with the telemonitoring device. It would be beneficial to clarify if their experiences (as compared to admitted patients) were different in terms of satisfaction or usability.
nakijken.**

This is an interesting remark. We checked our data on the experience of the telemonitorcohort in the 2 categories (admitted vs non-admitted) very roughly (we counted optimal scores (5 on a 5 point Likert scale) only). Although there are some differences regarding the experience with the system, these are not major. Because of the limited number of patients in this subanalysis and the data heaviness of the manuscript, we propose not to show these findings.

|  | **Admitted group**  **(N=9)**  **% optimal score** | **Non-admitted**  **group (N=12)**  **% optimal score** |
| --- | --- | --- |
| **Instruction ED clear** | 88 | 100 |
| **Insight into system** | 88 | 100 |
| **Plaster comfortable** | 88 | 58 |
| **Placement router easy** | 88 | 100 |
| **Satisfied with plaster** | 100 | 75 |
| **Satisfied with router** | 88 | 100 |
| **Satisfied with system** | 88 | 83 |
|  |  |  |
| **Had to learn much** | 100 | 100 |
| **Self confident in use** | 88 | 83 |
| **Simple system** | 88 | 100 |
| **Unnecessarily complex** | 11 | 0 |
| **Easy-to-use** | 88 | 100 |
| **Prefer self measurement** | 44 | 25 |
| **Alternative to admission** | 78 | 75 |
| **Reassuring effect** | 88 | 92 |
| **No problem privacy** | 100 | 100 |
| **Recommend to family** | 88 | 100 |
| **Use more often** | 88 | 100 |

• The results section is very data-heavy. Consider summarising main takeaways more narratively in paragraph form for better readability (especially around patient vs provider agreement).
We removed some of the data on agreement in order to relieve some of the heaviness of the data. We also moved data on the alerts to the supplemental data for the same reason.

We further deleted data on continued care (eg. Oxygen, infusion, Table 1) as well.

**• I recommend making the limitations section a distinct subheading under the Discussion. It would strengthen the manuscript to explicitly state that this was a convenience sample collected during office hours, which may affect generalisability to the full ED population, including evenings and weekends.**
Subheading added.

We mentioned this in the methods, and added the following to the discussion/limitations

“It is also possible that the proportion of patients willing and able to go home with home monitoring in evenings, nights and weekend differs from that during the office hours when this study was conducted”.

**• MCU/ICU explained well in text but define MEWS in full at first mention for clarity.**

Added to the methods section. **• In the Methods (Lines 85–86), please state the hospital’s location and country explicitly (e.g., “Maastricht University Medical Centre, Netherlands”). This provides helpful context for international readers.**

Added.

**Reviewer #4:**

**Thank you for the opportunity to review this study, "Telemonitoring at home for provision of continued care in the acute care setting. A prospective pilot study focusing on first experiences of patients". This pilot prospective study explored ED patients' and healthcare professionals' perspectives on home telemonitoring as an alternative to hospital admission. Eligible patients (adult, assessed by internists, and without exclusion criteria such as severe illness, confusion, or language barriers) completed questionnaires about their willingness and preferences for telemonitoring, and a subset was enrolled in an actual telemonitoring program if they lived within 15km of the hospital and were either discharged or admitted without need for continuous hospital monitoring.

While the topic of this study and the actual methodology and analysis seemed to be relevant and useful, the paper requires significant revision. Much of the need for review may stem from how language was used throughout the article, which can benefit from a thorough review by an English language expert. Additionally, the abstract needs to be reviewed thoroughly to include information that is later mentioned in the article, but missing from the abstract (e.g. the 3 questions being investigated in the research). Please see the following for detailed feedback.**

Thank you for your thorough review. We adjusted our manuscript to your comments. For details, see below. **=========================================================

In the abstract it's not clear what is meant by the percentages in the Abstract's Results section. Are authors giving percentages of patients who met the prerequisites for telemonitoring? If so, please clarify accordingly: "The prerequisites for telemonitoring were guaranteed admission if necessary (96.9%), possibility to contact the treatment team 24/7 (90.8%) and presence of someone to watch over the patient (72.4%)."**
We changed the text of the abstract to make this more clear to the readers. **----------------------------------------------------------

"Main barriers were: treatment could not be provided at home, feeling too ill, and judging it unsafe to return home" --> Barriers to what? Do authors mean barriers to being recruited for telemonitoring as part of the study (since a subset was started on telemonitoring)? Barriers that were identified by patients or doctors in a questionnaire?**
We meant barriers indicated by patients. The first paragraph of the abstract/results section was about the 98 patients anwering the questionnaire. We changed the abstract to make this more clear.

**----------------------------------------------------------

"In total, 11.2% of patients indicated that admission could be avoided using telemonitoring, while 6.1% thought this might be possible." --> If patients indicated that admission could be avoided did they also not think this might be possible? The distinction between these two groups of patients is unclear.**
We added the word “another” to make it more clear.  **----------------------------------------------------------

"agreement with patients was slight-fair" --> please indicate in the Methods section of the abstract how agreement was statistically ascertained.**
This analysis was further clarified in the analysis section.

“Regarding the agreement on possible avoidance of admission by using telemonitoring, Cohen’s kappa was calculated as well (only calculated in patients who were admitted to the hospital).” **----------------------------------------------------------

"Prerequisites were guaranteed admission and 24/7 contact with the treatment team..." --> Again, in the conclusion section of the Abstract, it's unclear what authors mean by "prerequisites". Do they mean "facilitators" as opposed to "barriers"?**
We asked the patients what was needed for them to be able to go home with telemonitoring. Probably, facilitator is a better word. Changed. **----------------------------------------------------------

"...barriers were related to disease severity and lack of someone to watch over the patient. Telemonitoring can be used in acute care as alternative for admissions if prerequisites are met and barriers removed." --> What do authors mean by "barriers removed"? How does one remove the barrier of disease severity? Do they mean if exclusion criteria are met?**
You are completely right. We removed the latter. **----------------------------------------------------------

In order to relieve the increasing pressure on the healthcare system and to adjust to
preferences of patients (1), alternative forms of care are being explored.
--> please actually give the magnitude of the pressure you are referencing (increasing ED usage over the years, worsening ED wait times, etc); please mention actual prefrences of patients so readers can know it aligns with the solution being presented here.**

We are afraid that we do not completely understand this comment, because we already added a reference on the positive attitude of patients on new care models (ref 1). We do not aim to reduce pressure on EDs by investigating new care models that start in the ED. In contrast, starting telemonitoring in the ED may even increase the workload, but this is beyond the scope of this manuscript.

**PLEASE REVIEW LANGUAGE / GRAMMAR:
- ABSTRACT: In this prospective pilot study, we asked medical ED patients on their perspectives on home monitoring. In a subset, we started and evaluated telemonitoring.**
You have made a number of suggestions regarding the language used in this manuscript. We carefully reviewed all your remarks and changed the text according to the suggestions you kindly provided. We also carefully reread the manuscript and corrected mistakes. For reasons of brevity of the response letter, we chose to not respond to each individual remark. **----------------------------------------------------------

- 63 To date, experience with telemonitoring in acute conditions in acute settings is scarce. --> ...telemonitoring of acute conditions...
----------------------------------------------------------
- 64 As alternative for admission, --> As an alternative to admission
----------------------------------------------------------
- 66 Telemonitoring at home turned out feasible and safe...--> Telemonitoring at home was shown to be feasible and safe; Please give citation.**

References were already provided in the first sentence on this topic (refs 6-9); we added the word COVID-19 to make it more clear that the statements were based on the reference we provided. **--------------------------------------------------------
- The sentences in lines 66-69 seem to be jumbled. Currently:
Telemonitoring at home turned out feasible and safe, and many patients could be treated 67 outside the hospital. However, there is limited experience with other acute conditions (10-14). The results were promising, and the patients recovered faster and moved more at home than patients who were admitted (13).
Did authors mean to say:**

**Telemonitoring at home turned out feasible and safe, and many patients could be treated outside the hospital. The results were promising, and the patients recovered faster and moved more at home than patients who were admitted (13). However, there is limited experience with other acute conditions (10-14)**

See our response on your last remark; this part of the paragraph could indeed be clearer; we adjusted the text to make it more clear that the first part of our statement concerned COVID-19 patients, and the last part patients with other acute conditions.

**“**However, there is limited experience with other acute conditions (10-14). The results of these studies were promising, and the patients recovered faster and moved more at home than patients who were admitted (13). “

**----------------------------------------------------------

- Unclear what authors are trying to say:
 Before home monitoring can be implemented on a larger scale, however, patient experience with home monitoring devices and their preferences should be further explored in the acute care setting, as are the ways to select patients who are suitable for and capable of using home monitoring (10, 15, 16)
Are they trying to say: Before home monitoring can be implemented on a larger scale, however, patient experience with home monitoring devices and their preferences should be further explored in the acute care setting, as [SHOULD BE METHODS] to select patients who are suitable for and capable of using home monitoring (10, 15, 16)**

We changed to text to make this more clear.

**“**Before home monitoring can be implemented on a larger scale, however, it is important to further investigate patient preferences and experiences with home monitoring devices in the acute care setting. Additionally, methods should be developed to identify patients who are both suitable for and capable of being monitored at home (10, 15, 16). “

**Please revise the wording here ("more information on these aspects"? This is a strange way to word what they might be trying to say)-->
74 If more information on these aspects becomes available, the necessary steps can be made to implement a new form of acute care starting in the ED**
We made this sentence more exact.

**“**If more information on preferences, experience and methods for identification of suitable candidates for homemonitoring becomes available, steps can be made..” **----------------------------------------------------------

What does "aimed to retrieve the proportion of patients..." mean? Do you mean "estimate the proportion of patients..."?
79 In addition, we aimed to retrieve the proportion of patients who could be treated at home instead of in the hospital by using telemonitoring**
Yes, we aimed to do that. We changed the text.

**----------------------------------------------------------
In the introduction section of the article (lines 66-81) the authors identify 3 parts to their investigation:
(1) investigate the preferences of ED patients regarding telemonitoring as continued care starting in the ED
(2) investigate the experiences of medical patients and acute care physicians with continuous telemonitoring started in the setting of an ED
(3) estimate "proportion of patients who could be treated at home instead of in the hospital by using telemonitoring"
The third goal of this paper is not mentioned in the abstract's methods.**
The third goal was added to the abstract:

“Suitability for homemonitoring was assessed by ED patients and care professionals.” **----------------------------------------------------------

109 about to be sent home --> about to be discharged home from the ED (not "about to be sent home").** Text changed **----------------------------------------------------------
What do you mean "aimed to retrieve"? "Telemonitoring concept"? Please revise this sentence:
112 because we aimed to retrieve
113 experiences of patients with the telemonitoring concept;**
We deleted the “aimed to retrieve..” part of the sentence because of redundancy.

**----------------------------------------------------------
What do you mean in the following sentence? Do you mean "the telemonitor cohort were asked to measure..."? Or do you mean that there are a group of telemonitor patients who did not return home such that you specified in the sentence "telemonitor patients who returned home"?
114 The telemonitor patients who returned home were asked to measure
 their vital signs (blood pressure, heartrate, oxygen saturation and temperature) three times a day**
The admitted patients did not have to measure their vital signs because nurses do so. We measured vital signs in a classical way (with “standard” devices to compare the vitals signs measured by the telemonitoring device with standard devices. The text is adjusted.

We meant to say: **“**The patients who were discharged home from the ED with the telemonitoring device were asked…”.  **----------------------------------------------------------

In the Methods section:
Unclear whether patients who returned home had actions that were required (i.e. asked to measure their vital signs as mentioned in lines 114 and 115 OR no actions were required from the patients as mentioned in lines 124 and 125):
114 The telemonitor patients who returned home were asked to measure
their vital signs (blood pressure, heartrate, oxygen saturation and temperature) three times a day.

VS

119 For this study, a telemonitoring device consisting of a plaster, applied to the chest, and a lead guided through the axilla of the non-dominant arm. The plaster and lead continuously measure the respiratory rate, heartrate, axilla temperature, and generate an output every 2 minutes. The thresholds of the vital signs and of the period during which the thresholds before an alarm was raised were agreed in advance. The data were transmitted by a portable router, which in turn transmitted the measurements and alarms via a web application. No actions were required from the patients**

You are right. We added ..”to use this telemonitoring device” to make this issue more clear. The telemonitoring device was very easy to use; just place the router correctly. No more actions were required. For the sake of the study, we asked the patients to measure vital signs theirselves as well (non-admitted patients).
**----------------------------------------------------------

From lines 119-125 it seems telemonitoring was happening in two places: at home for patients who were discharged from the ED; and in the hospital for patients who did not require continuous monitoring. Perhaps, revise the title, which indicates telemonitoring is happening at home: Telemonitoring at home for provision of continued care in the acute care setting. A prospective pilot study focusing on first experiences of patients.**
The aim was to collect the perspectives of patients on telemonitoring at home when they visited the ED with an acute medical problem, and to test the concept of telemonitoring starting in the setting of the ED. We tested this concept partlyin the hospital, but monitoring in the hospital was just a first step to find out whether the concept can be used in the home setting. This was mentioned in the aims of the study (Introduction).

We revised the title.

“Telemonitoring starting in the emergency department as alternative to acute hospital admission

A prospective pilot study focusing on patient preferences and first experience”

**----------------------------------------------------------

"...before an alarm was raised..."? Language needs to be revised to suit an academic journal.**

**122 The thresholds of the vital signs and of the period during which the thresholds
123 before an alarm was raised were agreed in advance.

Do you mean:** **"The thresholds for the vital signs, as well as the duration over which these thresholds had to be exceeded before an alarm was triggered, were determined in advance."
Similarly on line 299: "In total, 90 alarms were raised in 17 patients..." --> For an academic journal, alarms are "triggered"...not "raised".**

As Plos is indeed a US journal, we changed the verb used to trigger. **----------------------------------------------------------

Don't use "filled in" to refer to "completed":
133 on (17, 18)), and filled in by all participants... ---> COMPLETED by all participants
140 For the ED cohort, physicians and nurses filled out a short assessment questionnaire... --> physicians and nurses COMPLETED a short assessment questionnaire...**
Adapted

**----------------------------------------------------------

Throughout the article it seems the authors use the term "retrieve" quite frequently and incorrectly to indicate "collect" or "evaluate". They are using "retrieve" in a way that is atypical in the English language ("retrieve" is typically used to refer to fetching something that has been archived, not to refer to the collecting of or evaluate of data). Please revise this usage:**

Adjusted throughout the manuscript.
 **134 aimed to retrieve data on the availability of and skills regarding communication, medical and
135 digital instruments (e.g. blood pressure monitor and digital skills (18)). In addition,136 preferences were retrieved...
152 The experience with the telemonitoring system of the two acute internists was retrieved at the...
311 In this prospective pilot study, we retrieved perspectives...
330 ...it is important to retrieve the perspectives...
340 retrieved in qualitative studies...
345 It is important to retrieve whether this**

**348 Nursing care needs were not retrieved in this study.
Authors can use the term "retrieve" on line 155 (I think they may be getting "retrieve" and "collect" confused:
155 Electronic medical charts were used to collect data on age, sex, means of transportation to the
156 ED --> Electronic medical charts were used to retrieve data on age, sex, means of transportation to the ED...
----------------------------------------------------------
Please revise this sentence "between by patient" doesn't make sense:
168 Agreement between by patient, physician and nurse assessments of being “fully capable”
169 (physical, cognitive and home support) for telemonitoring and for avoiding admission.**
Typo has been removed.

**----------------------------------------------------------

Unsure what authors mean by "questions were asked on the judgement of their ability...":
137 Furthermore, questions were asked on the judgement of their ability to return home in their
138 current condition,**

We kindly refer to the questionnaires, provided in the supplemental material. We changed the text to make this topic more clear.

**“**Furthermore, the judgement of their ability to return home in their current condition was assessed, and, when applicable, on the reasons why returning home was not possible.”

**----------------------------------------------------------

It's not clear which, if any, of the outcomes listed in the primary and / or secondary outcomes list include the questionnaire data about patient perspectives on home monitoring.

162 Outcome measures
Primary**

**The proportion of patients satisfied with telemonitoring (telemonitor cohort)
Secondary
Proportion of patients willing and able to return home from the ED with telemonitoring as alternative for admission
Agreement between by patient, physician and nurse assessments of being “fully capable”
 (physical, cognitive and home support) for telemonitoring and for avoiding admission.
The number and nature of generated alarms.**

The primary endpoint concerns the proportion of patients satisfied with telemonitoring. Two paragraphs earlier, we refer to the *experience questionnaire* (suppl file 3), which we used to calculate this endpoint.

The secondary outcome (proportion of patients able to return home..) is answered using the *preference questionnaire* (patient view) and the *assessment questionnaire* (professional view). Both questionnaires are to be found in the supplementary files (1 and 2). The alarms were removed from the outcomes section for reasons of data heaviness (see below). **----------------------------------------------------------

Please fix the Table 1 so the "n" for the row about initial treatment after admission lines up with the columns; the way it's represented now is not clear which n belongs where: Initial treatment after admission (n=54;
n=9)**

After the ED visit, 54 of 98 patients were admitted to the hospital (in total 51 to a regular ward). Of the telemonitor cohort (n=21), 9 were admitted to the hospital. We are not sure what is not clear. However, we removed this part of the Table in response to other comments on data heaviness. **----------------------------------------------------------
Unclear how the authors are using the term "prerequisites". In some places it seems the authors are using the term "prerequisite" to refer to "preferences". In other places it seems they use the term "prerequisite" to refer to "patient stated requirement for telemonitoring" (which can be "patient prerequisite for telemonitoring"). Please clarify.**
We changed prerequites (in response to other comments) to facilitators, and we made sure the manuscript is more consistent now regarding to the chosen phrasing.

**229 The three most frequently mentioned prerequisites for telemonitoring were guaranteed admission if necessary (96.9%), possibility to contact the treatment team 24/7 (90.8%) and the presence of someone to watch over/assist the patient (72.4%).**

**Table 2: Prerequisites for telemonitoring
----------------------------------------------------------
271 In total, 21 patients used the telemonitor system. Their details are depicted in Table 1. --> Just cite the table: In total, 21 patients used the telemonitor system (Table 1).**

Adjusted **----------------------------------------------------------

For figures 2 and 3, please include 95% confidence intervals.**
It is not so easy to provide CIs if there are more than two categories. If there is just one category of interest, there is no problem with "binarizing" and extracting a CI for one proportion. However, “binarizing” cannot be done for each category, because it may cause problems as the numbers of responses for each category are dependent and multiple probability statements are made. The probability that each proportion lies in the interval could be very different than expected. We choose not to provide these CIs.

**----------------------------------------------------------

340 ...which involve patients in the setup... --> which involve patients in STUDY DESIGN**
adjusted **----------------------------------------------------------

359 These studies were positive – in terms of costs and safety. --> What do you mean "positive"? Please just state the effect: e.g. Studies have demonstrated safety and cost-effectiveness...
We chaged the text to be more exact.**

“These small studies showed that telemonitoring was cost-effective and safe” **----------------------------------------------------------

Do not need the second comma
359 Interestingly, patients treated in their homes, moved more than hospitalized patients. ---> Interestingly, patients treated in their homes moved more than hospitalized patients.**

Typo is removed.

Thank you for your thorough review and suggestions. **----------------------------------------------------------**
