# Supplementary material for: Telemonitoring starting in the emergency department as an alternative to acute hospital admission: A prospective pilot study focusing on patient preferences and first experience
Source: PLOS Digit Health. 2025 Jul 31;4(7):e0000962. doi: 10.1371/journal.pdig.0000962 (PMC12312925; doi:10.1371/journal.pdig.0000962)
Supplement: Attachment — Submitted filename: response to reviewers TM acute care Plos Digitalv3.docx [file pdig.0000962.s016.docx]

Dear editor and reviewers,

Thank you for your review of our revised manuscript (PDIG-D-25-00071R2; Telemonitoring starting in the emergency department as an alternative to acute hospital admission). We carefully revised the manuscript according to the comments made.

Please find our response below.

With kind regards,

on behalf of all authors.

Patricia Stassen

Dear Dr. Stassen,

Thank you for submitting your manuscript to PLOS Digital Health. After careful consideration, we feel that it has merit but does not fully meet PLOS Digital Health's publication criteria as it currently stands. Therefore, we invite you to submit a revised version of the manuscript that addresses the points raised during the review process.

[Note: HTML markup is below. Please do not edit.]

**Reviewers' Comments:**

Reviewer's Responses to Questions

**Comments to the Author**

1. If the authors have adequately addressed your comments raised in a previous round of review and you feel that this manuscript is now acceptable for publication, you may indicate that here to bypass the “Comments to the Author” section, enter your conflict of interest statement in the “Confidential to Editor” section, and submit your "Accept" recommendation.

Reviewer #4: All comments have been addressed

Reviewer #5: All comments have been addressed

2. Does this manuscript meet PLOS Digital Health’s [publication criteria](https://eur01.safelinks.protection.outlook.com/?url=https%3A%2F%2Ftrack.editorialmanager.com%2FCL0%2Fhttps%3A%252F%252Fjournals.plos.org%252Fdigitalhealth%252Fs%252Fjournal-information%2523loc-criteria-for-publication%2F1%2F010f019753cc55dd-d076dea1-bf91-4782-afd8-bd9b730f7a2d-000000%2FXLBymDMU501bhoMMPF5BvF50YJ_K3RjceimCFv6TxL8%3D214&data=05%7C02%7Cp.stassen%40mumc.nl%7C45237492e21b4519f51c08dda72f567d%7C08273b66221040cd8b205cf6cddc279a%7C0%7C0%7C638850543958480310%7CUnknown%7CTWFpbGZsb3d8eyJFbXB0eU1hcGkiOnRydWUsIlYiOiIwLjAuMDAwMCIsIlAiOiJXaW4zMiIsIkFOIjoiTWFpbCIsIldUIjoyfQ%3D%3D%7C0%7C%7C%7C&sdata=lKeJzMGkndb94htko8mquW65Yxy1hqu%2BdQcpSNeXnVM%3D&reserved=0)? Is the manuscript technically sound, and do the data support the conclusions? The manuscript must describe methodologically and ethically rigorous research with conclusions that are appropriately drawn based on the data presented.

Reviewer #4: Yes

Reviewer #5: Yes

3. Has the statistical analysis been performed appropriately and rigorously?

Reviewer #4: Yes

Reviewer #5: Yes

4. Have the authors made all data underlying the findings in their manuscript fully available (please refer to the Data Availability Statement at the start of the manuscript PDF file)?

The [PLOS Data policy](https://eur01.safelinks.protection.outlook.com/?url=https%3A%2F%2Ftrack.editorialmanager.com%2FCL0%2Fhttps%3A%252F%252Fjournals.plos.org%252Fdigitalhealth%252Fs%252Fdata-availability%2F1%2F010f019753cc55dd-d076dea1-bf91-4782-afd8-bd9b730f7a2d-000000%2FKDmhMfUYV-FoRC-tuVAS2qL49ygkLqFXBT8bSZaKFqg%3D214&data=05%7C02%7Cp.stassen%40mumc.nl%7C45237492e21b4519f51c08dda72f567d%7C08273b66221040cd8b205cf6cddc279a%7C0%7C0%7C638850543958498679%7CUnknown%7CTWFpbGZsb3d8eyJFbXB0eU1hcGkiOnRydWUsIlYiOiIwLjAuMDAwMCIsIlAiOiJXaW4zMiIsIkFOIjoiTWFpbCIsIldUIjoyfQ%3D%3D%7C0%7C%7C%7C&sdata=ku3tQov1s366fStx2D8z6S1342E6lkGFgAxLN3eij38%3D&reserved=0) requires authors to make all data underlying the findings described in their manuscript fully available without restriction, with rare exception. The data should be provided as part of the manuscript or its supporting information, or deposited to a public repository. For example, in addition to summary statistics, the data points behind means, medians and variance measures should be available. If there are restrictions on publicly sharing data—e.g. participant privacy or use of data from a third party—those must be specified.

Reviewer #4: Yes

Reviewer #5: Yes

5. Is the manuscript presented in an intelligible fashion and written in standard English?

PLOS Digital Health does not copyedit accepted manuscripts, so the language in submitted articles must be clear, correct, and unambiguous. Any typographical or grammatical errors should be corrected at revision, so please note any specific errors here.

Reviewer #4: Yes

Reviewer #5: Yes

6. Review Comments to the Author

Please use the space provided to explain your answers to the questions above. You may also include additional comments for the author, including concerns about dual publication, research ethics, or publication ethics. (Please upload your review as an attachment if it exceeds 20,000 characters)

Reviewer #4: Thank you for attempting to address my previous comments. Please conduct an English language review with someone who is a native speaker of the English language and who is not one of the co-authors. Once this is done I agree the manuscript is ready for publication. But please do the English language review.

Response: the manuscript has been checked.

Reviewer #5: Additional Suggested changes

Thank you for your suggestions below. The text has been adapted.

Abstract

Line 49. All telemonitored patients were satisfied with the easiness of use and comfortability of the system, which gave most patients a reassuring feeling and was considered an alternative to admission.
Suggested edits. All telemonitored patients were satisfied with the easiness ease of use and comfortability comfort of the system, which gave most patients a reassuring feeling reassurance and was considered an alternative to admission

Methods
Line 135. For this study, a telemonitoring device consisting of a plaster, applied to the chest, and a lead guided through the axilla of the non-dominant arm (17). The plaster and lead continuously measure the respiratory rate, heartrate, axilla temperature, and generate an output every minutes. The thresholds for the vital signs, as well as the duration over which these thresholds had to be exceeded before an alarm was triggered, were determined in advance. The data were transmitted by a portable router, which in turn transmitted the measurements and alarms via a web application. No other actions were required from the patients to use this telemonitor system.
Suggested edits: For this study, a wireless vital sign monitoring device (Sensium®, The Surgical Company, Abingdon, UK) was utilized. The system consists of a wearable patch applied to the chest, and a lead guided through the axilla of the non-dominant arm (17). The patch connected wirelessly to either hospital-based computers or via cellular connection to a mobile app to reveal vital sign history – i.e. respiratory rate, heart rate, axilla temperature, and changes over time. Thresholds for vital signs, as well as the duration over which these thresholds had to be exceeded before an alarm was triggered, were determined in advance. Once applied to the patient the system required no patient action on their part, transmitting data autonomously.

Line 194: Student T tests, Chi2 tests or Mann Whitney U tests,
Suggested edits: Student’s t-test, Chi-squared test or Mann-Whiteny tests
